# Intrinsic and extrinsic drivers of intraspecific variation in seed dispersal are diverse and pervasive

**DOI:** 10.1093/aobpla/plz067

**Published:** 2019-12-14

**Authors:** Eugene W Schupp, Rafal Zwolak, Landon R Jones, Rebecca S Snell, Noelle G Beckman, Clare Aslan, Brittany R Cavazos, Edu Effiom, Evan C Fricke, Flavia Montaño-Centellas, John Poulsen, Onja H Razafindratsima, Manette E Sandor, Katriona Shea

**Affiliations:** 1 Department of Wildland Resources and Ecology Center, Utah State University, Logan, UT, USA; 2 Department of Systematic Zoology, Adam Mickiewicz University, Poznań, Poland; 3 Department of Forestry and Natural Resources, Purdue University, West Lafayette, IN, USA; 4 Environmental and Plant Biology, Ohio University, Athens, OH, USA; 5 Department of Biology and Ecology Center, Utah State University, Logan, UT, USA; 6 Landscape Conservation Initiative, Northern Arizona University, Flagstaff, AZ, USA; 7 Department of Ecology, Evolution and Organismal Biology, Iowa State University, Ames, IA, USA; 8 REDD & Biodiversity Unit, Cross River State Forestry Commission, Calabar, Nigeria; 9 National Socio-Environmental Synthesis Center, University of Maryland, Annapolis, MD, USA; 10 Department of Wildlife Ecology and Conservation, University of Florida, Gainesville, FL, USA; 11 Nicholas School of the Environment, Duke University, Durham, NC, USA; 12 Department of Natural Resource Management, South Dakota State University, Brookings, SD, USA; 13 Department of Ecology, Evolution, and Environmental Biology, Columbia University, New York, NY, USA; 14 Center for Biodiversity and Conservation, American Museum of Natural History, New York, NY, USA; 15 Penn State University, University Park, PA, USA

**Keywords:** Crop size, fruit size, interindividual variation, intraindividual variation, seed dispersal effectiveness, seed dispersal traits

## Abstract

There is growing realization that intraspecific variation in seed dispersal can have important ecological and evolutionary consequences. However, we do not have a good understanding of the drivers or causes of intraspecific variation in dispersal, how strong an effect these drivers have, and how widespread they are across dispersal modes. As a first step to developing a better understanding, we present a broad, but not exhaustive, review of what is known about the drivers of intraspecific variation in seed dispersal, and what remains uncertain. We start by decomposing ‘drivers of intraspecific variation in seed dispersal’ into intrinsic drivers (i.e. variation in traits of individual plants) and extrinsic drivers (i.e. variation in ecological context). For intrinsic traits, we further decompose intraspecific variation into variation among individuals and variation of trait values within individuals. We then review our understanding of the major intrinsic and extrinsic drivers of intraspecific variation in seed dispersal, with an emphasis on variation among individuals. Crop size is the best-supported and best-understood intrinsic driver of variation across dispersal modes; overall, more seeds are dispersed as more seeds are produced, even in cases where per seed dispersal rates decline. Fruit/seed size is the second most widely studied intrinsic driver, and is also relevant to a broad range of seed dispersal modes. Remaining intrinsic drivers are poorly understood, and range from effects that are probably widespread, such as plant height, to drivers that are most likely sporadic, such as fruit or seed colour polymorphism. Primary extrinsic drivers of variation in seed dispersal include local environmental conditions and habitat structure. Finally, we present a selection of outstanding questions as a starting point to advance our understanding of individual variation in seed dispersal.

## Teaser

Intraspecific variation in the quantity and quality of seed dispersal has important ecological and evolutionary consequences, yet it is generally ignored in favour of simply using population mean values. In a broad but not exhaustive review we show that drivers of intraspecific variation in seed dispersal are diverse and pervasive. These include both intrinsic (i.e. variation in traits of individual plants) and extrinsic (i.e. variation in ecological context) drivers. Further, these drivers appear to interact frequently in complex ways. Current theory does not include or account for these complex and interacting drivers.

## Introduction

Intraspecific variation in seed dispersal has important consequences for individual reproductive success, plant population dynamics, community structure and evolution. For example, intraspecific variation in seed dispersal distances (Janzen 1970), the microhabitat destination of dispersed seeds (Schupp 1988) and the treatment in the mouth and gut (Traveset *et al.* 2007) affect demography and individual plant fitness through their impacts on the number of seeds dispersed, surviving, germinating and growing as seedlings. As a prominent example, dispersal kernels that include interindividual variation in dispersal distances are not equal to a population-level dispersal kernel based on mean dispersal distances. Including this intraspecific variation can alter the rate of population spread and the extent of gene flow (Schreiber and Beckman 2019; Wyse *et al.* 2019). Furthermore, individual variation in seed dispersal increases the range of habitats and conditions where seeds are dispersed, increasing the likelihood of the population to persist under unfavourable events (the portfolio effect; Bolnick *et al.* 2011). Although poorly studied, intraspecific variation in seed dispersal may also influence community assembly, species richness and responses to anthropogenic changes (Snell *et al.* 2019). See Snell *et al.* (2019) for a thorough review of the consequences of intraspecific variation in dispersal. However, given the historical focus in seed dispersal studies on population means, there are large gaps in our understanding of intraspecific variation in dispersal. We do not know how pervasive detectable variation in seed dispersal is, what the drivers of individual variation are and to what extent drivers have independent versus interactive effects. To date there only have been scattered efforts to summarize the breadth of our understanding of the drivers of intraspecific variation in seed dispersal.

The phrase ‘intraspecific variation in the drivers of seed dispersal’ is diffuse and subsumes multiple types of drivers and levels of variation. Decomposing this variation helps structure our thinking about intraspecific variation in dispersal. First, drivers of intraspecific variation in seed dispersal can be categorized as intrinsic variation based on trait expression of individual plants (e.g. fruit crop size, seed size, plant height) and extrinsic variation based on the ecological context of the plant (e.g. fruiting neighbourhood, topography). Further, intraspecific variation can be divided into variation among individuals (interindividual variation) and variation within individuals (intraindividual variation) (Herrera 2017). Most drivers of intraspecific variation in seed dispersal have both an interindividual and an intraindividual component (e.g. fruit size, fruit sugar concentration; even crop size or fruiting neighbourhood of the same individuals vary over time).

When considering drivers of intraspecific variation in seed dispersal, it is important to clarify what aspects and consequences of dispersal are being affected. Seed dispersal effectiveness, or SDE, depends on both the quantity of seeds dispersed (i.e. the immediate outcome of dispersal) and the quality of dispersal provided to those seeds (i.e. the delayed consequences of dispersal; Schupp 1993; Schupp *et al.* 2010, 2017; reviewed in Box 1). While SDE is usually viewed as mean quantity multiplied by mean quality, these means are derived from a sample of individuals that likely differ substantially in both the quantity and the quality of dispersal. Beyond SDE, the probability of long-distance dispersal (LDD) can vary intraspecifically, which in turn contributes to population spread and gene flow. In this review, we focus mostly on seed movement, largely because that is what we have the most information on. However, we address consequences for seedling establishment or recruitment where relevant information is available.

In this paper, we provide a broad but not exhaustive review of the drivers of intraspecific variation in the quantity, and to a lesser extent, the quality components of seed dispersal (see Table 1 for a summary). We emphasize intrinsic drivers and interindividual variation because of our interest in individual fitness, defined as the contribution of an individual to future generations (Sæther and Engen 2015) (see Herrera 2009, 2017 for a focus on intraindividual variation in plant traits). However, we also consider intraindividual variation in traits because it can scale up to affect interindividual variation in dispersal. Further, intraindividual variation is not independent of interindividual variation. Lastly, we consider simple intraspecific variation in traits because much relevant work focuses on population-level trait variation without considering its apportionment into intra- and interindividual components.

**Table 1.**
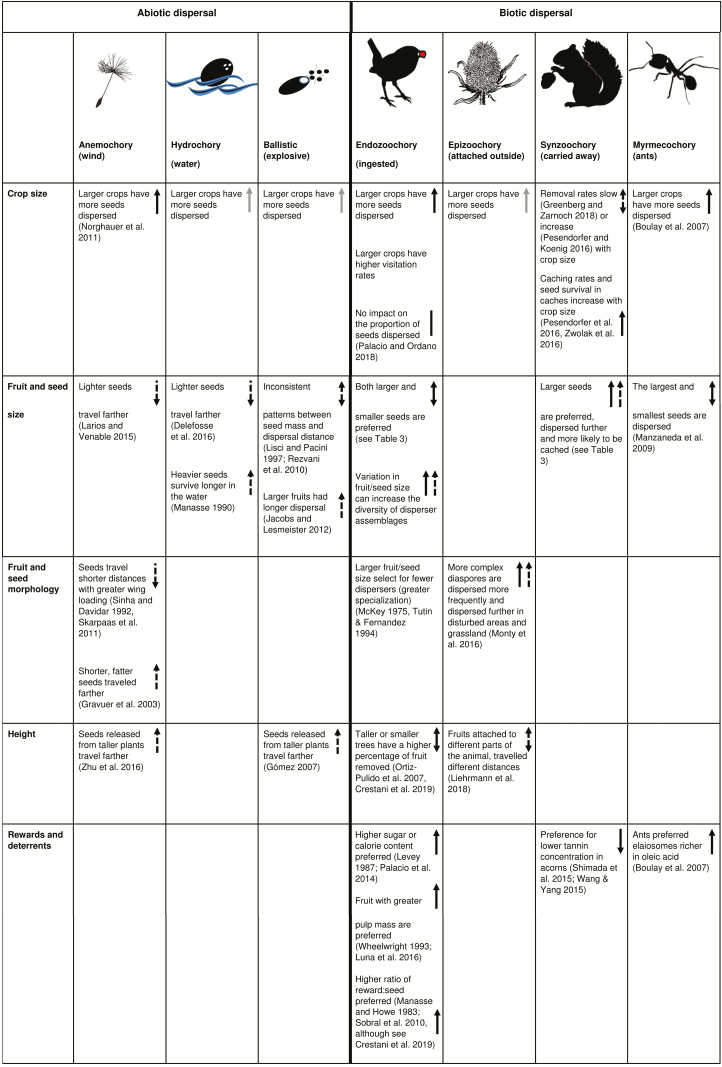
An overview of how intrinsic variation in plant traits influences seed dispersal quantity and quality. Quantity is indicated by solid arrows, while dashed arrows are for quality. Grey arrows indicate uncertainty (i.e. we assume this relationship to be true but no studies have explicitly measured this), and vertical lines without arrowheads indicate a lack of relationship. Representative references are included, however this is not meant to be an exhaustive list. See text for more details.

We have several goals with this review. First, we illustrate the breadth of drivers of interindividual variation in seed dispersal. Second, we use diverse examples to illustrate the broad geographic and taxonomic scope of interindividual differences in seed dispersal, to assess how consistently they occur and to explore the range of impacts on seed dispersal processes. Third, we briefly discuss the barriers to fully understanding these drivers and their effects.

## Intrinsic Variation: Plant Traits

### Fruit crop size

Crop size (i.e. seed production) varies substantially among individuals and populations within a season and across years (e.g. Norghauer *et al.* 2011; Norghauer and Newbery 2015). Crop size is probably the most widely studied and best-supported driver of interindividual variation in the quantity of seeds dispersed. A strong positive relationship between crop size and the number of seeds dispersed and in the number of seeds being dispersed long distance is expected in abiotic dispersal modes such as anemochory and hydrochory and in the biotic dispersal mode epizoochory (Clark *et al.* 1998; Table 1). However, the expectation is less clear with endozoochory, synzoochory and myrmecochory, where animal dispersers make foraging decisions in resource-heterogeneous environments where these dispersers can be satiated (e.g. Manasse and Howe 1983; Hampe 2008; Table 1) or prematurely leave feeding trees in order to mix diets with complementary resources (Whelan *et al.* 1998; Morán-López *et al.* 2018a). Nonetheless, expected patterns with respect to crop size have been proposed for endozoochory. Howe and Estabrook (1977) developed two models based on specialized (model 1) versus opportunistic (model 2) frugivore/seed dispersal systems. They suggested that the number of seeds dispersed should increase with fruit availability for both types of species, although the number dispersed should plateau for model 1 plant species that depend on specialized dispersers that tend to involve relatively few species and become satiated. They further predicted that the effect of crop size on the proportion of the available seeds dispersed would differ for model 1 and model 2 species. For model 1 species, the proportion of seeds dispersed was expected to initially increase with crop size but would reach a peak at some intermediate crop size due to disperser satiation and then drop with ever larger fruit crops. In contrast, for model 2 species they predicted that the proportion of the seed crop dispersed would increase with increasing crop size, perhaps stabilizing at a constant proportion at larger crop sizes, but not decreasing. However, the dichotomy between specialized and opportunistic dispersal systems is not generally accepted at this point (Schupp *et al.* 2010), leading other authors (e.g. Carlo *et al.* 2007) to propose a general expectation that the number of seeds dispersed should increase with increasing crop size. In fact, this is considered one major driver of the development of frugivory hubs, where hub individuals in the network (those with the largest fruit crops) receive more dispersal services than expected, leaving non-hub individuals with little dispersal services (Carlo *et al.* 2007).

This last prediction appears to be supported by studies mostly of endozoochory that demonstrate that as crop size increases, visitation rate by avian (e.g. Saracco *et al.* 2005; Ortiz-Pulido *et al.* 2007; Guerra *et al.* 2017) and mammalian (e.g. Guitián and Munilla 2010) dispersers increases, which translates into an increased quantity of seeds dispersed (Table 2). For example, *Prunus mahaleb* fruit crop size explained 80 % of seeds dispersed in a population in southern Spain (Jordano and Schupp 2000). With respect to the proportion of seeds dispersed, results to date show no consistent relationship (Table 2). These patterns suggesting a general increase in the number but not the proportion of seeds dispersed with increasing crop size are supported by a meta-analysis that found positive bird-mediated selection on fruit crop sizes as measured by both visitation rate and the quantity of seeds dispersed, but no selection on the proportion of seeds dispersed (Palacio and Ordano 2018).

**Table 2.** Examples of studies reporting the relationships between interindividual variation in plant crop size and both the number of seeds removed (#) and the proportion of the seed crop removed (prop.). Arrows denote shape of the relationship between crop size and the variable, with a dot representing no information.

Species	Form of the relationship with increasing crop size		Reference
	# seeds removed	Prop. seeds removed	
Tropical endozoochorous tree			
* Casearia corymbosa*	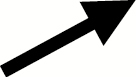	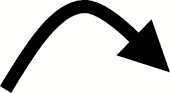	Ortiz-Pulido *et al.* (2007) (#), Howe and Vande Kerckhove (1979) (prop.)
* Eugenia uniflora*	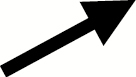	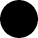	Blendinger and Villegas 2011
* Guarea glabra*	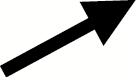	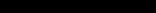	Howe and De Steven (1979)
* Virola nobilis*	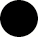	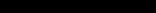	Manasse and Howe (1983)
* Virola surinamensis*	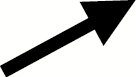	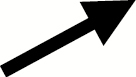	Moreira *et al.* (2017)
Tropical endozoochorous shrub			
* Erythroxylum havanense*	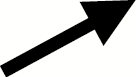	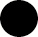	Gryj and Domínguez (1996)
* Miconia fosteri*	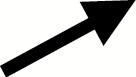	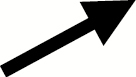	Blendinger *et al.* (2008)
* Miconia irwinii*	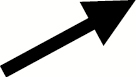	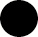	Guerra *et al.* (2017)
* Miconia serrulata*	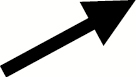	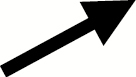	Blendinger *et al.* (2008)
Temperate endozoochorous tree			
* Olea europaea*	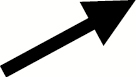	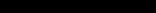	Alcántara *et al.* (1997)
* Prunus mahaleb*	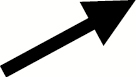	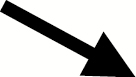	Jordano (1995)
Temperate endozoochorous shrub			
* Crataegus monogyna*	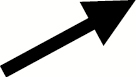	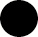	Sallabanks (1993)
* Prunus virginiana*	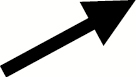	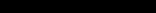	Parciak (2002)
* Sambucus pubens*	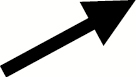	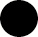	Denslow (1987)
Temperate synzoochorous tree			
* Quercus lobata*	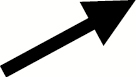	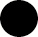	Pesendorfer and Koenig (2016)

Crop size can also affect the quality component of SDE and the probability of LDD. Increasing crop size lifts the entire dispersal kernel, resulting in more seeds in the tail of the distribution and thus more LDD and increased population spread and gene flow (Clark *et al.* 1998). Increasing crop size also results in more seeds dispersing farther in a local dispersal context, increasing the chances of reaching suitable sites (Norghauer *et al.* 2011) and surviving distance- and density-dependent mortality (Janzen 1970; Connell 1971). Although empirical evidence is limited, crop size can also affect the quality of endozoochorous dispersal by altering disperser behaviour and disperser assemblages of individual plants. For example, as *Vassobia breviflora* crop size increased, disperser residence time in the canopy decreased, increasing the probability of seed dispersal away from the parent rather than seeds processed *in situ* (Palacio *et al.* 2017). Increasing crop size also increased fruit consumption by legitimate dispersers (gulpers) without affecting consumption by pulp consumers, altering the realized dispersal assemblage and increasing overall dispersal quality (Palacio *et al.* 2017). Lastly, with a population-wide increase in *Fagus sylvatica* crop size (masting), there was an increase in survival of seeds cached by *Apodemus flavicollis*, a clear increase in the quality of dispersal (Zwolak *et al.* 2016; Table 1). On the other hand, seed survival in caches can be lower under trees that produced large seed crops (Schubert *et al.* 2018); thus, spatial variation in crop size might have different effects than temporal variation (masting).

### Fruit and seed size

Fruit and seed size variation is likely the second most widely studied driver of interindividual variation in seed dispersal. Fruit and seed size vary within individuals, but also among individuals, years and populations (Sobral *et al.* 2013; González-Varo and Traveset 2016; Herrera 2017). In a study of 39 species from 46 populations, on average 62 % of seed size variation was within individuals while 38 % was among individuals, though individual species varied substantially (Michaels *et al.* 1988). Thus, fruit and seed size variation can influence animal disperser decisions regarding which plants to forage in (interindividual) and then which fruits to consume (intraindividual) (Vanthomme *et al.* 2010; Effiom *et al.* 2013). Furthermore, mean fruit size of individuals can be highly heritable, indicating potential selection response (Wheelwright 1993; Galetti *et al.* 2013).

Many studies have demonstrated size-based fruit or seed selection by dispersers, suggesting a potentially important role for fruit/seed size in driving interindividual variation in the quantity component of SDE, although actual patterns of selection are not consistent and appear to depend on the plant and animal species involved (Table 3a). It is generally thought that fruit/seed size-based selection is a function not so much of fruit/seed size, but rather by the fruit/seed size relative to the disperser size. For example, for endozoochorous birds that swallow fruits whole, it is widely believed that fruit selection is driven by fruit diameter and bird gape width (e.g. Wheelwright 1993; González-Varo and Traveset 2016). Similarly, it is thought that seed size selection by synzoochorous seed dispersers is related to the ratio of seed to disperser size (Muñoz and Bonal 2008a).

**Table 3.** Examples of studies on fruit or seed size selection by animal dispersers. In endozoochory (a) selection always denotes preferential removal/dispersal. In synzoochory (b) we consider the relationship between seed size and various quantitative (removal/dispersal) and qualitative (consumption, caching %, caching distance, seedling production) metrics of SDE. For myrmecochory (c), the only stuy of which we are w=aware, is presented as in endozoochory (a).

Plant species	Animal dispersers	Selection for	Reference
(a) Endozoochory			
*Corema album*	*Oryctolagus cuniculus*	Smaller	Larrinaga (2010)
*Viburnum opulus*	*Erithacus rubecula*, *Turdus philomelos*	Smaller	Hernández (2009)
*Prunus mahaleb*	Birds	Smaller	Jordano (1995)
*Prunus virginiana*	Birds	Smaller	Parciak (2002)
*Virola nobilis*	Birds	Smaller	Howe and Vande Kerckhove (1981)
*Crataegus monogyna*	*Turdus migratorius*	Larger	Sallabanks (1993)
*Crataegus monogyna*	*Turdus* spp.	Larger	Martíinez *et al.* (2007)
*Ocotea tenera*	Birds	Larger	Wheelwright (1993)
*Henriettea succosa*	Birds	Larger	Crestani *et al.* (2019)
*Olea europaea* var*. sylvestris*	Birds	Larger (1 of 2 years)	Alcántara *et al.* (1997)
(b) Synzoochory			
*Quercus ilex*	*Garrulus glandarius*	Trees with smaller acorns, but larger individual acorns (removal, one of two habitats)	Morán-López *et al.* (2015a)
*Carapa procera*	*Myoprocta acouchy*	Larger (removal, caching %, and caching distance)	Jansen *et al.* (2004)
Myrcianthes coquimbensis	Rodents	Larger (removal and caching %)	Luna *et al.* (2016)
*Quercus rubra*	Rodents	Larger (caching %)	Wróbel and Zwolak (2017)
*Pinus armandii*	*Apodemus latronum* and *Apodemus chevrieri*	Larger (removal, consumption, and caching %)	Wang and Ives (2017)
*Astrocaryum mexicanum*	*Heteromys desmarestianus*	Larger (consumption); no effect (caching); smaller (caching distance, cache survival)	Brewer (2001)
*Quercus ilex*	Rodents	No effect (removal); larger (caching %, caching distance, cache survival)	Gómez *et al.* (2008)
*Quercus serrata*	Rodents	No effect (removal and caching); larger (caching distance, cache survival)	Xiao *et al.* (2004)
*Quercus ilex*	*Apodemus sylvaticus* and *Mus spretus*	No effect (removal by *A. sylvaticus*); smaller (removal by *M. spretus*)	Muñoz and Bonal (2008a)
*Quercus robur*	*Garrulus glandarius*	Intermediate-sized (removal)	Bossema (1979)
*Carapa oreophila*	Rodents	No effect (removal, consumption, caching %, and caching distance)	Yadok *et al.* (2018)
*Pittosporopsis kerrii*	Rodents	Larger (removal); intermediate (caching %, caching distance, seedling production)	Cao *et al.* (2016)
(c) Myrmecochory			
*Helleborus foetidus*	Ants	Smaller and larger (removal)	Manzaneda *et al.* (2009)

The extent to which fruit/seed size selection contributes to interindividual differences in the quantity of seeds dispersed is unclear. Dispersers may select among individual plants based on mean traits or among individual fruits independent of the mother plant. While some studies demonstrate that frugivores select among fruiting plants based on mean fruit or seed size (e.g. Howe and Vande Kerckhove 1981; Wheelwright 1993; Alcántara *et al.* 1997; Martínez *et al.* 2007), others demonstrate that at the population level, individual fruits are selected based on their sizes (e.g. Parciak 2002; Hernández 2009; Larrinaga 2010). Thus, even strong selection of fruits based on size need not lead to differential selection of individual plants based on fruit size. For example, dispersers of *P. mahaleb* strongly selected fruits based on size, but this was almost entirely driven by selection of smaller fruits within an individual plant’s fruit crop, while there was inconsistent and weak selection among individual plants based on fruit size (Jordano 1995). Because multiple traits associated with selection by dispersers may be correlated with seed size, the degree to which selection is driven by fruit or seed size, rather than a correlated trait is unclear (Jordano 1984; Martínez *et al.* 2007). For example, the four main avian dispersers of *Rubus ulmifolius* in southern Spain differed in the distribution of seed sizes dispersed, but seed size, seed number, pulp/seed ratio and percent pulp co-varied, making it difficult to determine which trait or traits were being selected (Jordano 1984).

Fruit and seed size can also drive intraspecific variation in the quantity and quality of dispersal in other ways. For gape-limited, endozoochorous birds, intra- and interindividual variation in fruit diameter can affect the proportion of a plant’s fruit crop that a disperser can swallow. For instance, in a *Myrtus communis* population in southern Spain, some individuals produced large fruits that only *Turdus merula* and *T. philomelos* could swallow and disperse. However, other individuals in the same population produced smaller fruits that were completely available to these species and partially available to *Sylvia atricapilla*, *Erithacus rubecula* and, in the case of one individual, the smallest disperser, *S. melanocephala* (González-Varo and Traveset 2016). Thus, the realized disperser assemblages of individual plants varied from two to five species. Moreover, realized disperser assemblages of individual plants varied across years due to changes in fruit size.

Such among-individual and among-year variation in realized disperser assemblages can affect interindividual variation in dispersal outcomes. First, variation in the number of animal species feeding on an individual plant likely affects the quantity of seeds dispersed (Schupp *et al.* 2010). Second, interindividual variation in realized disperser assemblages is expected to drive interindividual variation in LDD, gene flow and the quality of dispersal because disperser species differ in their dispersal kernels, treatment in the mouth and gut, and microhabitat destination of seeds (Jordano and Schupp 2000; García *et al.* 2007; Schupp *et al.* 2010). Species-specific preferences in microhabitat and fruit/seed size can also result in microhabitats accumulating different seed size distributions (Obeso *et al.* 2011). Lastly, seed size affects whether a seed is swallowed and passed through the digestive system versus being dropped, spat out or regurgitated, which affects both treatment in the mouth and gut and dispersal distances (Corlett and Lucas 1990; Jordano 1992; Kunz and Linsenmair 2008).

Fruit and seed size also affect synzoochorous and myrmecochorous dispersal (Table 3b). In general, larger seeds tend to be dispersed more rapidly and farther, and are more likely to be cached than smaller seeds; in contrast, no obvious pattern links seed size and the probability of surviving in a cache. The actual outcome of the interaction may be more related to the ratio of seed to disperser size rather than seed size alone (Muñoz and Bonal 2008a). However, three species of rodents varying 4-fold in mass all preferentially selected and dispersed larger fruits of the Chilean desert shrub *Myrcianthes coquimbensis* (Luna *et al.* 2016).

While there is abundant evidence that synzoochorous dispersers select and handle individual seeds based on size, there are fewer studies documenting dispersers selecting on mean seed size among individual plants. The large Japanese wood mouse (*Apodemus speciosus*) preferentially dispersed seeds of individual *Q. serrata* trees with larger mean acorn size (Shimada *et al.* 2015). Similarly, *Apodemus* spp. disproportionately dispersed and cached seeds from *Pinus armandii* individuals with larger mean seed mass (Wang and Ives 2017), although the greater probability of their seeds being consumed cancelled the benefits of increased dispersal. By contrast, mean seed size of the Queen palm (*Syagrus romanzoffiana*) had no influence on tree selection by squirrels (Alves *et al.* 2018), and *Garrulus glandarius* preferentially fed on *Quercus ilex* trees with smaller acorns (Morán-López *et al.* 2015a). Thus, although evidence is limited, there is potential for seed size to contribute to interindividual variation in the quantity and quality of seed dispersal by synzoochorous dispersers.

Although the consequences of within-individual variation in plant traits have not been considered frequently in ecology (e.g. Herrera 2017), in addition to selection based on individual or mean fruit/seed size, mutualistic dispersers may select among individual plants based on the extent of intraindividual variation in fruit or seed size. In a latitudinal study of *Crataegus monogyna* seed dispersal by *Turdus* spp. in Europe, birds selected against intraindividual fruit size variation in populations with lower variation and selected for intraindividual fruit size variation in populations with higher variation (Sobral *et al.* 2013). Similarly, *A. speciosus* not only selected individual *Q. serrata* trees with larger acorns, but also selected individual trees with a greater variability in acorn weight (Shimada *et al.* 2015).

Seed size also affects abiotic seed dispersal. In the seagrass *Zostera marina*, settling rate increases with seed size, suggesting smaller seeds disperse farther (Delefosse *et al.* 2016). When grazed by the specialist herbivore *Ophraella communa*, the riparian weed *Ambrosia artemisiifolia* produces lighter, more buoyant seeds, demonstrating a clear mechanism for interindividual variation in dispersal (Fukano *et al.* 2014). In ballistically dispersed species, both seed and fruit size can affect patterns of seed dispersal. In *Oxalis acetosella* (Berg 2000) and *O. corniculata* (Rezvani *et al.* 2010), dispersal distances increased with seed mass, while in *Mercurialis annua*, dispersal distances decreased with increasing seed mass (Lisci and Pacini 1997). In the only study on fruit size and ballistic dispersal of which we are aware, dispersal distance increased with fruit length in *Erodium cicutarium* (Jacobs and Lesmeister 2012).

It is generally believed that dispersal distances of anemochoric species will decrease as seed mass increases, and this expectation appears to be well-supported, although the variance explained is generally low. This general pattern has been reported in both tropical and temperate environments as well as across trees, shrubs and herbaceous species (Morse and Schmitt 1985; Bhuyan *et al.* 2000; Meyer and Carlson 2001; Bullock *et al.* 2003; Debain *et al.* 2003; Gravuer *et al.* 2003; Skarpass *et al.* 2011), although there are exceptions (e.g. Wyse *et al.* 2019). Given that seed mass varies both among and within individuals (Sinha and Davidar 1992; Gravuer *et al.* 2003), seed mass variation may contribute to interindividual variation in dispersal distances. For example, under highly competitive conditions, plants of the wind-dispersed desert annual *Dithyrea californica* produce smaller, lighter seeds that are dispersed farther (Larios and Venable 2015). Given the typical heterogeneous distribution of individuals in populations, it is likely that *D. californica* individuals vary continuously in competitive environments and thus potentially in dispersal ability. Finally, the actual pattern of wind dispersal is driven not simply by seed mass, but by the relationship between seed mass and the dispersal structure (e.g. pappus, Skarpaas *et al.* 2011; and see below under Morphology).

### Height: plant, seed abscission, seed attachment

Interspecific studies of the effect of plant height on seed dispersal suggest plant height is a major correlate of dispersal distances and is considerably more important than seed size (Thomson *et al.* 2011, 2018; Augspurger *et al.* 2017). However, given that height of reproductive adults varies substantially more among than within species, it need not follow that the more limited interindividual variation in plant height will be a major driver of interindividual variation in seed dispersal distances. Nonetheless, limited empirical evidence suggests that interindividual height variation might be at least a minor driver of interindividual variation in dispersal, at least for abiotically dispersed species. With anemochory, plant (or seed release) height has been shown to be positively related to dispersal distances in trees (Sinha and Davidar 1992) and herbaceous perennials (Sheldon and Burrows 1973; Weiblen and Thomson 1995; Skarpass *et al.* 2011; Zhang *et al.* 2011; Zhu *et al.* 2016; DiTommaso *et al.* 2018), in some cases relatively strongly (e.g. Zhu *et al.* 2016). In *Carduus nutans*, height, and therefore dispersal potential, is environmentally plastic, increasing with simulated climate change (Zhang *et al.* 2011). Interestingly, increasing tree height in *Lophopetalum wightianum* not only led to greater dispersal distances and larger seed shadows, but also more even seed dispersion, potentially decreasing density-dependent mortality (Sinha and Davidar 1992). Norghauer *et al.* (2011) suggest that tree height does not affect dispersal distances of mahogany in Amazonian forests because all reproductive trees are emergent above the canopy and exposed to winds. Note, however, that much of our understanding comes from controlled releases of seeds at fixed heights in wind tunnels, and that the heterogeneity of the real world likely reduces the explanatory power of plant height. Intraindividual variation in seed release height could further obscure any potential interindividual variation in dispersal distances based on variation in plant height. Interindividual height variation is also important for ballistic dispersal. Increasing height resulted in increasing dispersal distances in the mustards *Erysimum mediohispanicum* (Gómez 2007), *Arabidopsis thaliana* (Wender *et al.* 2005) and *Lepidium campestre* (Thiede and Augspurger 1996), but not in *O. acetosella* (Berg 2000).

We have less direct evidence that height is important for biotic dispersal. Interspecific studies suggest that seed presentation height can affect the frequency of epizoochorous dispersal (Hughes *et al.* 1994; Fischer *et al.* 1996; Graae 2002; Wessels *et al.* 2008; Hovstad *et al.* 2009; Albert *et al.* 2015), but data for intraspecific effects of height variation are limited. In two studies on small tropical trees, variation in interindividual plant height affected endozoochorous seed dispersal; in *Casearia corymbosa*, plant height very weakly affected fruit removal (Ortiz-Pulido *et al.* 2007), and in *Henrietta succosa*, dispersers favoured shorter individuals (Crestani *et al.* 2019). Other studies have documented vertical segregation of frugivore communities in tropical forests (Shanahan and Compton 2001; Poulsen *et al.* 2002; Lahann 2007; Yoshikawa *et al.* 2009; Flörchinger *et al.* 2010), so fruiting conspecifics of different heights may have at least marginally different disperser assemblages, which could affect both the quantity and quality of dispersal.

### Rewards and deterrents

Effects of variation in rewards and deterrents on seed dispersal have been studied extensively, though mostly from the perspective of interspecific differences (e.g. Cazetta *et al.* 2008 for frugivorous bird-dispersed trees; Vander Wall 2010 for rodent-cached trees); effects of intraspecific variation have received much less attention. Nonetheless, available data suggest that interindividual variation in rewards and deterrents may be an important driver of interindividual variation in seed dispersal in some cases.

Intraspecific variation in fleshy fruit seed dispersal driven by intraspecific variation in rewards offered to animal dispersers has been addressed in a variety of ways with some studies focusing on the quality of the reward, and some on the absolute or relative quantity of reward. Interspecific comparisons show that fruit colour is predictive of protein (brightness), sugar (chroma) and lipid (darkness and chroma) concentrations (Cazetta *et al.* 2012; Schaefer *et al.* 2014), and that these signals can be effective even if relationships are weak (Albrecht *et al.* 2018). We have long known of interindividual variation in fruit sugar content and that dispersers can distinguish these differences by taste (Levey 1987). However, it is not known whether dispersers can distinguish intraspecific differences in nutrients based on colour. Nonetheless, dispersers distinguish among plants that differ in rewards in some manner (Crestani *et al.* 2019). In China, the frequency with which the deer *Muntiacus muntjak* visited fruiting *Choerospondias axillaris* trees was correlated with mean Kcal/fruit (Chen *et al.* 2001). Similarly, birds selected for *Celtis ehrenbergiana* trees (Palacio *et al.* 2014) and *Sambucus pubens* shrubs (Denslow 1987) with greater mean sugar concentration of fruits. However, frugivores preferentially fed on *Henriettea succosa* individuals with intermediate sugar concentrations (Crestani *et al.* 2019). These limited results suggest that variation in fruit energy content is a potential driver of interindividual variation in seed dispersal. We are aware of no evidence that endozoochorous dispersers discriminate among plants based on variation in other nutrients. Individual *Virola nobilis* trees varied substantially in protein, lipid and non-structural carbohydrate content, but this variation did not explain variation among trees in seed dispersal (Manasse and Howe 1983).

There is more extensive evidence that either the absolute or relative quantity of reward is important in fruiting plant selection by dispersers. As noted previously, independent of body size, three rodent species preferentially selected larger fruits of the shrub *M. coquimbensis*; larger fruits had more pulp, and it was the pulp, not the seed, that was consumed (Luna *et al.* 2016). In *Ocotea tenera*, dispersers selected trees with larger diameter fruits, as noted above, apparently due to the greater pulp mass; fruit diameter explained much of the variation in total pulp mass (*r*^2^ = 0.56) but not in the ratio of seed mass/fruit mass (*r*^2^ = 0.01) (Wheelwright 1993). However, more typical are studies demonstrating selection driven by intraspecific variation in relative rather than absolute reward per fruit. In *V. nobilis* (Howe and Vande Kerckhove 1981; Manasse and Howe 1983), *V. calophylla* (Russo 2003) and *C. monogyna* (Sallabanks 1993; Martínez *et al.* 2007; Sobral *et al.* 2010), variation in dispersal was explained in part by mean pulp:seed ratio but not by the absolute quantity of pulp. Sometimes this resulted in the selection of smaller fruits (Howe and Vande Kerckhove 1981), sometimes the selection of larger fruits (Martínez *et al.* 2007; Sobral *et al.* 2010).

Such interactions between fruit size and either pulp quantity or pulp:seed ratio suggest caution when considering the role of fruit or seed size variation in interindividual variation in seed dispersal. As noted, many studies report fruit selection based on fruit or seed size, and size clearly has an impact on seed dispersal, at a minimum by filtering which dispersers can swallow and disperse the seeds of endozoochorously dispersed species. However, in many cases where dispersers select larger or smaller fruits, we do not know if it is selection based on fruit/seed size or rather selection based on a correlated trait such as absolute or relative quantity of the reward.

Most work on deterrents with fleshy-fruited plants has focused on hypotheses addressing why ripe fruits contain toxins rather than assessing interindividual variation in concentrations and dispersal. In the single field study of which we are aware, mean emodin concentration in *Rhamnus alaternus* fruit pulp was unrelated to fruit removal rate among plant individuals in 1 year but was positively correlated with removal rate in another year (Tsahar *et al.* 2002). Levey and Cipollini (1998) showed that cedar waxwings (*Bombycilla cedrorum*) feeding on artificial fruits discriminated against ‘fruits’ containing realistic concentrations of α-solamargine compared to ‘fruits’ without this glycoalkaloid, but did not discriminate among artificial fruits that differed in α-solamargine concentration. However, potential seed disperser species differ in sensitivity to varying tannin concentrations in artificial fruits (Zungu and Downs 2015). Whitehead and Poveda (2011) reported a potential environmental influence on intraspecific variation in deterrents; in *Hamelia patens*, artificial herbivory of subtending leaves reduced fruit removal in adjacent inflorescences due to reduced palatability, presumably a result of herbivory-induced chemical changes. Plant secondary compounds also can alter gut retention times of seeds, which can affect seed dispersal distances and germination (Tewksbury *et al.* 2008; Baldwin and Whitehead 2015), although we know little about the degree of natural intraspecific variation, particularly interindividual variation, in secondary compounds, and the consequences of this variation.

In myrmecochorous species, chemical composition of the elaiosome has been studied mostly through interspecific comparisons of seed and elaiosome chemical profiles (e.g. Fischer *et al.* 2008). Fatty acid composition in *Euphorbia characias* elaiosomes, especially of oleic acid, varies among populations and among individuals within populations, but not within individuals (Boieiroa *et al.* 2012). In the related *Helleborus foetidus*, this variation is ecologically important (Boulay *et al.* 2007) as seed-dispersing ants preferentially visited plants with elaiosomes richer in oleic acid. Many consider oleic acid to be an attractant or behavioural trigger rather than a reward because elaiosomes are rich in other critical nutrients, such as amino acids (Fischer *et al.* 2008). However, it is likely both attractant and reward since it evokes seed harvesting and it is the biosynthetic precursor of the essential nutrients linoleic and linolenic acids (Fischer *et al.* 2008).

In synzoochorous dispersal systems, evidence presented above that these dispersers frequently preferentially harvest, disperse, disperse farther or cache larger seeds reflects a response to the reward offered since the seed is both the propagule being dispersed and the reward offered for dispersal (Gómez *et al.* 2019). However, whether it is seed size itself or energy content that drives the decision is unclear. A study with artificial seeds suggests the answer is complicated in that energy was the primary predictor of initial harvest, but size was the primary determinant of post-harvest fate (removed versus *in situ* consumption, distance dispersed, and cached versus consumed after dispersal; Wang and Yang 2014).

Variation in seed defences can simultaneously affect the risk of seed predation and the probability of seed dispersal. A particularly well-studied example of this phenomenon involves mechanical seed defences in synzoochorous limber pine (*Pinus flexilis*). Cone structure in this species is under a conflicting selective pressure from the red squirrel (*Tamiasciurus hudsonicus*), a predispersal seed predator, and the Clark’s nutcracker, a seed disperser (Siepielski and Benkman 2010). Individuals that produce particularly well-defended cones tend to be dispersed by *Peromyscus* mice rather than the Clark’s nutcracker (Siepielski and Benkman 2008), resulting in much shorter dispersal distances and different habitat destinations of the seeds.

Endocarp or seed coat thickness as a deterrent has long been considered an evolutionary response to synzoochory, in particular to managing the dual role of seed dispersal and seed predation, and there is substantial interspecific support for the idea that these dispersers select and handle seeds at least partly based on endocarp or seed coat thickness (see Vander Wall 2010). Intraspecific studies on the effects of seed coat thickness are sparse and inconsistent; mean endocarp thickness of the palm *S. romanzoffiana* did not influence tree selection by the squirrel *Guerlinguetus ingrami* (Alves *et al.* 2018), but *P. flexilis* seeds with thicker seed coats were more likely to be cached by rodents and were dispersed further than seeds with thinner coats (Siepielski and Benkman 2008). Chemical deterrents may also play a role in interindividual dispersal by synzoochorous dispersers. Mice preferred individual *Quercus serrata* trees producing acorns with lower mean and coefficient of variation of tannin concentration (Shimada *et al.* 2015). Similarly, rodents were less likely to remove acorns produced by *Q. rubra* trees from nitrogen-addition plots, presumably due to changes in chemical composition (Bogdziewicz *et al.* 2017).

Interpretation of these results is complicated by frequent co-variation of size, nutritional content and concentration of secondary metabolites in fruits and seeds (e.g. Izhaki *et al.* 2002), as discussed above with co-variation of fruit size and absolute or relative quantity of reward in fleshy-fruited plants. Interestingly, co-variation of these traits can even vary with position of fruits in the plant canopy (Houle *et al.* 2007). Recent innovative work with artificial fruits has begun to tease apart the disparate roles of size, nutrients and secondary compounds in seed selection by caching rodents. Wang and Yang (2015) manipulated seed size, tannin, fat, protein and starch content in artificial seeds and showed how all these factors affected rodent foraging. Rodents preferentially removed seeds with less tannin; increasing fats, and to a lesser degree, proteins, reduced this negative effect. Seed size, tannins and nutrient content of artificial seeds all affected various stages of the seed dispersal process by rodents, with size and nutrients tending to favour dispersal, and tannins disfavouring dispersal (Wang *et al.* 2013). In contrast, artificial seed experiments with *Dasyprocta punctata* in Costa Rica suggest size but not tannin concentration affect seed dispersal decisions (Kuprewicz and García-Robledo 2019). Furthermore, artificial seeds with different characteristics were cached in different microhabitats, which can affect quality of dispersal; larger and more nutritious seeds were most likely to be cached under shrubs (Wang and Corlett 2017).

### Morphology

Here we consider forms of morphological variation beyond fruit and seed size that might influence interindividual variation in dispersal. The most apparent cases of morphology driving interindividual variation in seed dispersal are with heterocarpic species that produce diaspores of two or more distinct morphologies differing in dispersal ability. For example, in the annual grass *Bromus tectorum* plants produce caryopses with (complex) and without (simple) sterile florets attached, which differ in dispersal ability because complex diaspores attach better to animal fur (Monty *et al.* 2016). Numerous plant species produce dimorphic, soft (non-dormant) and hard (dormant) seeds (Baskin and Baskin 2014). Paulsen *et al.* (2013, 2014) argued that this strategy enables individual plants to benefit both from the antipredation advantages of hard seeds and the dispersal advantages of soft seeds. Hard seeds emit fewer volatiles than soft seeds and are more difficult to detect by granivores such as rodents that rely on olfaction. Because granivores act both as dispersers and predators, detection might be advantageous, but can also result in seed consumption. In heterocarpic species, the relative proportions of different propagule types often vary with environmental conditions. For example, increasing stress can result in either an increase (Imbert and Ronce 2001; Martorell and Martínez-López 2014) or a decrease (Mandák and Pyšek 1999; Lu *et al.* 2013) in the production of more dispersible morphs. Interestingly, *Calendula arvensis* produce three distinct fruit morphs, one adapted to epizoochory, one to anemochory and one without adaptations for dispersal, although the extent of interindividual variation in the production of different morphs is unknown (De Clavijo 2005).

Beyond simply fruit or seed size, actual dimensions or shape can influence foraging decisions of endozoochorous and synzoochorous dispersers. For example, fruit diameter independent of length or overall mass is thought to be the most important metric of fruit size for frugivorous birds based on how fruits are swallowed (e.g. Wheelwright 1993). Acorn shape also influences preference by the European jay (*G. glandarius*); when diameter was held constant, jays preferred longer acorns; when length was constant, they chose wider acorns; and when mass was constant, they chose longer and slimmer acorns over shorter and wider acorns (Bossema 1979). How widespread such patterns are is unknown.

As noted previously, patterns of anemochorous dispersal are not driven solely by seed mass, but are influenced by the relationship between seed mass and the dispersal structure, which varies intraspecifically. It is generally thought that wing (coma, pappus) loading, frequently measured as fruit mass per unit surface area of the dispersal structure, is the major determinant of dispersal ability in wind-dispersed species. Thus, in trees (Sinha and Davidar 1992; Bhuyan *et al.* 2000; Debain *et al.* 2003), shrubs (Meyer and Carlson 2001) and forbs (Morse and Schmitt 1985; Donohue 1998; Skarpaas *et al.* 2011), increasing wing loading results in shorter dispersal distances or greater falling velocities, implying shorter dispersal distances. Other traits of propagules also lead to intraspecific variation in wind dispersal. Sheldon and Burrows (1973) argued that the fine details of pappus architecture influence dispersal more than wing loading. In *Zygophyllum xanthoxylon*, a ‘shape’ index explained a small portion of the variation in dispersal distance (Zhu *et al.* 2016). For *Liatris scariosa* var. *novae-angliae*, dispersal distances were influenced by achene length (negatively), achene width (positively) and pappus length (positively), all of which differed among populations and among maternal families within populations, indicating likely interindividual variation in seed dispersal distances (Gravuer *et al.* 2003). *Carduus nutans* respond plastically to experimental drought with decreased wing loading and terminal velocity due to reductions in seed mass without changes in plume characteristics; interestingly, they also showed decreased intraindividual variation in terminal velocity (Teller *et al.* 2014).

The role of morphological variation in seed dispersal potential for other dispersal modes is poorly understood. In epizoochory, morphological variation has been examined almost exclusively interspecifically (e.g. Tackenberg *et al.* 2006; Will *et al.* 2007; Hovstad *et al.* 2009; Albert *et al.* 2015). However, limited evidence exists that seeds of epizoochorous species also vary intraspecifically in number, size and shape of appendages and in attachment potential (Gorb and Gorb 2002). We are aware of only one relevant study on hydrochory, where intraspecific variation in the size of the aeriferous mesocarp layers in *Scaevola crassifolia* fruits affected buoyancy (Guja *et al.* 2014).

### Colour polymorphism

Some fleshy-fruited species exhibit fruit colour polymorphism, producing two or more colour morphs, sometimes on different plants and sometimes on the same plant (e.g. Willson and O’Dowd 1989). Selection of particular colour morphs ranges from relatively strong (e.g. *Rubus spectabilis*, Gervais *et al.* 1999) to weak (e.g. *Rhagodia parabolica*, Willson and O’Dowd 1989) to non-existent (e.g. *M. communis*, Traveset *et al.* 2001). Selection can be consistent across large geographic areas (e.g. *R. spectabilis*, Gervais *et al.* 1999) or vary across years and populations (e.g. *Acacia ligulata*, Whitney 2005) or even among individuals of a disperser species (e.g. various birds dispersing *R. spectabilis*, Traveset and Willson 1998). In the only study we know addressing selection among individual plants, the deer *M. muntjak* preferred *C. axillaris* trees with yellow fruits over those producing yellowish-green fruits (Chen *et al.* 2001). The basis of colour morph selection is unclear, but there is no evidence we are aware of that in colour polymorphic systems morphs differ in size, pulp:seed ratio or major nutrients (Willson and O’Dowd 1989; Traveset and Willson 1998).

### Phenology

Interindividual variation in fruiting phenology is widespread in herbaceous forbs (Collier and Rogstad 2004), shrubs (SanMartin-Gajardo and Morellato 2003) and trees (Howe and Vande Kerckhove 1979; Franklin and Bach 2006; Muhanguzi and Ipulet 2012), but this variation may or may not affect seed dispersal. In *C. corymbosa*, later fruiting trees were visited by more species, but this had no real effect on dispersal because the additional frugivore species ate very few fruits (Howe and Vande Kerckhove 1979). *Olea europaea* var. *sylvestris* individuals that ripened fruit earlier were favoured in 1 of 2 years (Alcántara *et al.* 1997), while *O. tenera* trees ripening fruits earlier had greater and more rapid fruit removal than late-ripening trees (Wheelwright 1993). In *Q. serrata*, acorns produced later in the season were larger with lower tannin concentrations, making them more valuable food items (Takahashi *et al.* 2011). Phenological variation potentially affects dispersal quality as well. Although not linked to individual plant fruiting phenology, González-Varo *et al.* (2019) demonstrated that dispersal quantity and quality changed through the fruiting season of the bird-dispersed *Pistacia lentiscus*.

## Extrinsic Variation: Ecological Context

### Fruiting neighbourhood

The presence, species identity, density and relative desirability of co-occurring fruiting neighbours can influence interindividual variation in seed dispersal. Some argue that trees compete with neighbours for dispersal and that intraspecific competition should be more intense than interspecific competition, especially in the tropics where it was thought there was little overlap in dispersal assemblages across species (Howe and Estabrook 1977). However, facilitation of dispersal by neighbours is also a possibility if the collective lure of multiple fruiting trees disproportionately attracts dispersers (Morales *et al.* 2012). In fact, this scenario has been proposed as another driver of hub and non-hub dispersal networks since frugivores are assumed to choose high-quality patches to forage in without considering the number of trees contributing to that patch (Carlo *et al.* 2007).

As expected, intraspecific competition has been found to reduce the quantity of dispersal of many tropical and temperate trees and shrubs (Table 4). However, many studies also have found intraspecific facilitation of the quantity of seed dispersal across taxa and ecosystems (Table 4). Thus, both intraspecific competition and facilitation have been found to affect the quantity of dispersal in both tropical and temperate systems, but given the relative scarcity of empirical work and its bias to tropical systems, general patterns are not clear. In a multispecies comparison, species fruiting in high densities were more likely to have dispersal reduced by neighbours (competition), whereas species fruiting in low density were more likely to have dispersal increased by neighbours (facilitation), a logical expectation (Albrecht *et al.* 2015). Further, whether competition or facilitation of dispersal by conspecific fruiting neighbourhoods occurs can be affected by the heterospecific fruiting neighbourhood (Rumeu *et al.* 2019).

**Table 4.** Examples of studies reporting intraspecific competition for dispersers (i.e. reduction in dispersal quantity by conspecific neighbours), intraspecific facilitation of dispersal (i.e. increase in dispersal caused by conspecific neighbours) or no effect of neighbours including a variety of life forms in tropical and temperate regions.

Species	Description	Result	Reference
*Schefflera morototoni*	Tropical tree	Competition	Saracco *et al.* (2005)
*Virola nobilis*	Tropical tree	Competition	Manasse and Howe (1983)
*Virola surinamensis*	Tropical tree	Competition	Moreira *et al.* (2017)
*Erythroxylum havanense*	Tropical shrub	Competition	Gryj and Domínguez (1996)
*Attalea butyracea*	Tropical palm	Competition	Jansen *et al.* (2014)
*Sambucus pubens*	Temperate shrub	Competition	Denslow (1987)
*Viburnum recognitum*	Temperate shrub	Competition	Smith and McWilliams (2014)
*Viburnum dentatum*	Temperate shrub	Competition	Smith and McWilliams (2014)
*Eugenia uniflora*	Tropical tree	Facilitation	Blendinger and Villegas (2011)
*Miconia fosteri*	Tropical shrub	Facilitation	Blendiger *et al.* (2008)
*Miconia irwinii*	Tropical shrub	Facilitation	Guerra *et al.* (2017)
*Geonoma pauciflora*	Tropical palm	Facilitation	Pizo and Almeida-Neto (2009)
*Tristerix corymbosus*	Temperate mistletoe	Facilitation	Morales *et al.* (2012)
*Juniperus communis*	Temperate shrub	Facilitation	García *et al.* (2001)
*Quercus ilex*	Temperate tree	Facilitation	Morán-López *et al.* (2015a)
*Miconia serrulata*	Tropical shrub	No effect	Blendiger *et al.* (2008)

Heterospecific fruiting neighbourhoods might also influence interindividual variation in dispersal quantity and quality, although we have even less empirical evidence for heterospecific than conspecific interactions, and outcomes appear to be complex. Dispersal of the tropical tree *Eugenia uniflora* was unaffected by heterospecific fruiting neighbourhoods (Blendinger and Villegas 2011). Similarly, *Solanum americanum* in monospecific patches and in mixed patches with *Cestrum diurnum* did not differ in the quantity of seeds dispersed; however, in the presence of *C. diurnum*, *S. americanum* seeds were dispersed in smaller seed loads among more defecations, resulting in reduced potential competition and increased number of sites occupied (Carlo 2005). In the south-eastern USA, the native shrub *Morella cerifera* marginally facilitated the dispersal of the invasive shrub *Triadica sebifera* and improved its germination, but inhibited seedling growth (Battaglia *et al.* 2009). In California oak woodlands, the high-quality disperser California scrub-jay (*Aphelocoma californica*) responded numerically and functionally to *Quercus lobata* with large acorn crops when the dominant *Q. douglasii* had low acorn production, but not when *Q. douglasii* produced abundant acorns (Pesendorfer and Koenig 2017). By contrast, the seed predator acorn woodpecker (*Melanerpes formicivorus*) had a constant response to *Q. lobata* trees independent of background acorn production. Consequently, *Q. lobata* trees received high-quality dispersal when *Q. douglasii* acorns were sparse, but little dispersal and extensive seed predation when *Q. douglasii* acorns were abundant. Synzoochorous foragers collect seeds both for current consumption and future use, but preferences often differ between consumed and cached items (Lichti *et al.* 2017), opening the potential for complex, indirect seed–seed interactions. For example, one seed species could provide a preferred short-time food supply and therefore subsidize caching of another species more suitable for long-term storage. Such ‘apparent predation’ (*sensu*Lichti *et al.* 2014) was documented between *Quercus robur* and *Q. rubra* in Poland (Bogdziewicz *et al.* 2019).

Finally, fruiting neighbourhoods can affect dispersal quality as well as quantity. With both endozoochorous and synzoochorous dispersal, higher density fruiting neighbourhoods have been shown to result in shorter dispersal distances (Carlo and Morales 2008; Morales *et al.* 2012; Jansen *et al.* 2014).

### Habitat structure: broader aspects

Beyond fruiting neighbourhoods, effects of other aspects of habitat structure on seed dispersal have been addressed from local within-patch variation in structure to landscape-scale variation. Here we give a brief overview from the perspective of interindividual variation in seed dispersal, emphasizing smaller scale population-level variation, with scale defined by the dispersal agent. Note that the drivers and consequences of habitat effects on interindividual variation in seed dispersal operate at much larger spatial scales for plants that are dispersed by mobile animals than for those dispersed abiotically.

At the scale of meters to tens of meters, the distances of *P. mahaleb* individuals from nests and rock outcrops affected the composition of the avian disperser assemblages foraging on those plants (Fuentes *et al.* 2001). Similarly, but at a larger spatial scale, three nearby stands with vegetation differing in vertical structure and species composition differed substantially in seed disperser assemblages foraging on *P. mahaleb* (Guitián *et al.* 1992). In both cases, differences in assemblages resulted in differences in the quantity of dispersal, the handling of fruits and seeds, and the microhabitat destination of dispersed seeds. In a highly heterogeneous forest, *C. monogyna* individuals growing with dense tree cover dispersed more seeds and over longer distances than did individuals growing with more sparse cover (Herrera *et al.* 2011). Similarly, with greater amount and continuity of forest cover, the carnivore *Martes foina* dispersed seeds longer distances (Herrera *et al.* 2016). Lastly, *Corema album* seeds were dispersed by the same three species in three adjacent habitat patches varying in vegetation structure, but all three species exhibited among-habitat variation in both the quantity and quality of dispersal (Calviño-Cancela and Martín-Herrero 2009).

More discrete habitat patchiness can also drive interindividual variation in dispersal quantity and quality. In *O. europaea* var. *sylvestris*, genetic information on avian dispersers and seed parents revealed major differences in dispersal for trees in remnant forest stands versus isolated trees in adjacent agricultural fields (González-Varo *et al.* 2017), with forest and isolated trees differing substantially in the assemblage of birds dispersing their seeds and in the destinations of dispersed seeds. In continuous forest, *G. glandarius* dispersed more *Q. ilex* acorns, dispersed them further, and cached them in better microhabitats than when foraging in adjacent open dehesas with only scattered oaks, while within dehesas, trees close to forest or in spatial clumps were more likely to be dispersed (Morán-López *et al.* 2015a).

Numerous studies have addressed habitat fragmentation effects on seed dispersal. In the Amazonian tree *Duckeodendron cestroides*, dispersed by arboreal and terrestrial mammals, both the quantity and the mean and maximum distance of dispersal were greater in continuous forest than in fragments (Cramer *et al.* 2007). Similarly, for the bird-dispersed African tree *Leptonychia usambarensis*, compared to continuous forest, fragments had fewer species and individuals of seed dispersers, had fewer seeds removed and had seedlings located closer to parents (Cordeiro and Howe 2003). Fragmentation combined with hunting led to the loss of larger-gaped dispersers and a reduction in seed dispersal of larger fruits, resulting in rapid evolution of reduced fruit and seed size in *Euterpe edulis* (Galetti *et al.* 2013). Fragmentation can also impact synzoochorous dispersal. In *Astrocaryum aculeatum*, decreasing forest patch area was associated with a higher quantity of dispersal (increased seed removal rate), but lower quality of dispersal (reduced caching and reduced dispersal distances), likely due to changes in rodent community composition (Jorge and Howe 2009). Similar findings have been found in *Q. ilex* dispersed by *Apodemus sylvaticus* (Morán-López *et al.* 2015b, 2018b). However, fragmentation does not always negatively affect seed dispersal; forest fragmentation in Poland reduced the number of larger frugivores without decreasing fruit removal (Farwig *et al.* 2017).

A meta-analysis of primarily tropical fleshy-fruited species (Markl *et al.* 2012) suggested that fragmentation does not affect visitation or seed removal rates, and only marginally reduces dispersal distances. By contrast, a meta-analysis of a worldwide data set (Fontúrbel *et al.* 2015) suggested that fragmentation reduced interaction rates (visitation or fruit removal), but not disperser diversity (abundance or species richness); at a major biome level, fragmentation reduced disperser diversity only in temperate zones but reduced interaction rates in both temperate and tropical zones. Additionally, inter- or intraspecific variation in disperser traits such as movement distance, movement frequency and gut retention time of seeds represent one mechanism explaining how fragmentation can positively or negatively affect dispersal distances (fragment entrapment, Jones *et al.* 2017).

Beyond fragmentation, habitat disturbance, degradation and simplification can impact dispersal quantity and quality. In oaks (*Quercus velutina* and *Q. alba*), timber harvest resulted in 67 % reduction in SDE by rodents, probably due to increased vegetation cover facilitating recovery of cached acorns (Kellner *et al.* 2016). In a Mediterranean system, habitat degradation reduced the abundance, species richness and movement of avian dispersers, resulting in reduced fruit removal, seed dispersal distances, seed survival and seedling success (Rey and Alcántara 2014). Other studies have shown that increasing forest disturbance can result in decreased likelihood of plants being visited by dispersers (Lehouck *et al.* 2009; Moreira *et al.* 2017), as well as decreased species richness of dispersers and reduced dispersal distances (Chatterjee and Basu 2015) and of rates of seed dispersal (Lehouck *et al.* 2009). These results are compatible with meta-analyses showing degradation to have a greater negative impact than fragmentation on seed dispersal (Markl *et al.* 2012), and habitat degradation generally reducing abundance and diversity of dispersers (Fontúrbel *et al.* 2015).

Habitat structure also can impact seed dispersal by wind, by altering the wind speed that initiates seed release, and by damping wind speeds. In *Taeniatherum caput-medusae* and *Tragopogon dubius*, taller surrounding vegetation reduced dispersal distances (Davies and Sheley 2007). Modelling suggests this should be common in herbaceous communities (Soons *et al.* 2004). Modelling further suggests that forest canopy height heterogeneity influences the likelihood of LDD; seeds released over shorter parts of the canopy encounter greater turbulence and are more likely to be ejected and experience LDD (Bohrer *et al.* 2008). Lastly, accelerated seed dispersal by wind along linear disturbances in the Canadian oil sands region has been reported (Roberts *et al.* 2018).

### Topography

Movements of frugivorous birds are influenced by subtle variation in topographic relief, which can affect which individual fruiting trees are encountered during foraging and where seeds are deposited (Westcott 1994, 1997); this is likely true for other animal vectors as well. However, little empirical work directly addresses the role of topography in interindividual variation in seed dispersal. In Ecuador, contrasting results were found for two fleshy-fruited shrub species (Blendinger *et al.* 2008); *Miconia fosteri* on ridges had a greater number and proportion of fruits removed than did those at the bottom of slopes, while *M. serrulata* had a greater number but not proportion of fruits removed on slopes than on either ridges or at the bottom of slopes. For wind dispersal, modelling suggests that even moderate topographic variability can have large impacts on variation in dispersal distances and directionality (Trakhtenbrot *et al.* 2014). Finally, slope steepness influences dispersal distances of heavy seeds, which are more likely to roll down downhill (e.g. oak acorns, Ohsawa *et al.* 2007), and the likelihood of seed dispersal via run-off (De Rouw *et al.* 2018).

### Non-disperser animal communities

Individual plants might also differ in the quantity and quality of animal-mediated seed dispersal due to the actions of third-party players. Predators can indirectly affect seed dispersal through their effects on risk-sensitive foraging of dispersers. Some of these effects are mediated by vegetation structure, with plants in more open and risky places receiving fewer visits by dispersers (Iida 2004; McCabe and Olsen 2015; Kellner *et al.* 2016). In other situations, animals respond to olfactory, visual or acoustic predator cues, leading to reduced seed removal rates in frugivorous birds (Breviglieri and Romero 2016; Tella *et al.* 2016; Shave *et al.* 2018), bats (Breviglieri *et al.* 2013) and granivorous rodents (Sunyer *et al.* 2013). In addition, rodents are sensitive to ungulate presence because of trampling risk or disturbance by rooting (e.g. by wild boar *Sus scrofa*); in *Q. ilex*, the presence of ungulates was associated with lower quality seed dispersal by rodents (lower proportion of seeds cached and not recovered) and changes in caching sites (reduced caching under shrubs) (Muñoz and Bonal 2007). Finally, responses to predators and competitors can interact with other traits, such as the presence and concentration of deterrents (McArthur *et al.* 2012).

Insects frequently infest fruit pulp, seeds or dispersal structures, which can affect seed dispersal. Howler monkeys (*Alouatta caraya*) preferentially feed in *Ocotea diospyrifolia* trees with high fruit infestation by curculionids and low fruit infestation by moths (Bravo 2012). The seed parasitoid wasp *Macrodasyceras hirsutum* reduces attractiveness of *Ilex integra* berries to frugivorous birds through ‘colour manipulation’; infested fruits are less likely to ripen and turn red, decreasing the risk that the fruits will be eaten and wasps killed (Takagi *et al.* 2012). In synzoochorous dispersal, seed infestation can increase the probability of rejection (Bossema 1979; Muñoz and Bonal 2008b) or of immediate consumption (Steele *et al.* 1996; Perea *et al.* 2012), but generally reduces caching rates (Steele *et al.* 1996; Perea *et al.* 2012), thus decreasing dispersal quality. However, not all scatter hoarders discriminate between infested and sound seeds, particularly before insect emergence (Gálvez and Jansen 2007; Cheng and Zhang 2011). Note that these synzoochorous examples are based on responses to individual seeds and it is unknown to what degree they translate into selection among trees differing in infestation levels. Insect attack also affects anemochory. For example, *Rhinocyllus conicus* larvae feeding on *C. nutans* receptacles induce callus formation, resulting in inhibited seed release, shortened pappus filaments and reduced dispersal distances (Marchetto *et al.* 2014). In turn, insect infestation is often affected by masting (Espelta *et al.* 2008, but see Bogdziewicz *et al.* 2018), thereby creating another, indirect pathway through which temporal variation in seed output can affect seed dispersal.

## Lifetime Fitness: Temporal Complexity

Most of what we know about intraspecific variation in seed dispersal represents a snapshot in time—a frame or two in a potentially long movie of life. While these frames might accurately represent the fitness outcomes for an annual plant, the majority of plants discussed in this review are long-lived perennials that are interacting with an extremely dynamic world where both intrinsic and extrinsic factors vary through time. Although we are not in a position to evaluate the overall consequences of this variation, it is important to acknowledge the variation exists.

Fruit crop sizes vary between years. Sometimes this variation is relatively subtle and driven by such factors as resource availability or climatic conditions (Jordano 1987; Jordano and Schupp 2000; Wenk and Falster 2015; Davi *et al.* 2016). Sometimes the variation is extreme, as seen in masting species (Herrera *et al.* 1994; Wenk and Falster 2015; Davi *et al.* 2016; Pearse *et al.* 2016). Different dispersal kernels are necessary to capture mast versus non-mast years (Martínez and González-Taboada 2009), with potentially greater LDD when acorn density is low (Moran and Clark 2012). The fitness impacts of this variation should depend at least partially on how synchronous fruit crop size variation is in the population and community. Fruit crop size also varies over longer, ontogenetic time scales; crop sizes increase with perennial plant age and size, often plateauing at some point and remaining relatively constant until death, sometimes showing declines with senescence late in life (Davi *et al.* 2016).

Many other intrinsic traits relevant to intraspecific variation in dispersal are temporally dynamic. Fruit/seed size, and most likely such traits as pulp:seed ratio, vary across years (González-Varo and Traveset 2016). Plant height increases ontogenetically (Coopman *et al.* 2008). Rewards (Lotan and Izhaki 2013) and deterrents (Tsahar *et al.* 2002) can change from year to year and in some cases even seasonally.

Temporal variation in extrinsic factors, or the ecological context, is perhaps even more extreme. Fruiting neighbourhoods can change from year to year as different individuals and species respond differently to changing resources and climate (Jordano and Schupp 2000). Other aspects of habitat structure around individual plants can change through time due to successional processes and demographic processes (Herrera *et al.* 1994), as well as anthropogenic impacts (Markl *et al.* 2012; Fontúrbel *et al.* 2015). Lastly, interactions with non-disperser animal communities can vary greatly from year to year as a function of, among other drivers, changes in individual crop sizes and in fruiting neighbourhoods, and population fluctuations of other interacting animal species (Schmidt and Ostfeld 2003).

## Complexity

Although many exceptions exist, much work on intraspecific variation in seed dispersal has taken a more or less univariate approach; for example, the impact of fruit crop size, fruit size or plant height on dispersal. Alternatively, some address multiple traits affecting dispersal and quantify the relative importance of each and the presence or absence of interactions. In a recent study using an individual-plant-based network analysis of frugivory, locations of individual *H. succosa* trees within the network were determined by a combination of plant height, fruit size and sugar concentration, with shorter individuals with larger fruits and intermediate sugar concentration being most central (Crestani *et al.* 2019). Nonetheless, the true complexity of dispersal is often overlooked. In this review we have also taken primarily a univariate approach, which we argue has value, especially at our early stage of understanding the drivers of interindividual variation in seed dispersal. However, it is critical to understand that we do not believe that this is really how the world exists.

We noted the difficulties of knowing what animal seed dispersers base their harvesting decisions on when so many potentially important traits co-vary: fruit size, absolute and relative quantity of reward, seed number and size, nutrients, toxins and more (Jordano 1984; Izhaki *et al.* 2002). For example, do frugivores select fruits to harvest based on size *per se* or on the underlying variation in pulp:seed ratio (Howe and Vande Kerckhove 1981; Martínez *et al.* 2007; Sobral *et al.* 2010)? Such complexities surely exist in other dispersal systems as well. For example, in anemochorous plants, the size of the dispersal structure increases with seed mass, but generally not sufficiently to maintain a constant wing loading (Meyer and Carlson 2001; Debain 2003; Skarpaas *et al.* 2011; but see Wyse *et al.* 2019). Co-variation of seed release height and seed terminal velocity (Teller *et al.* 2014), and of abscission force and terminal velocity (Teller *et al.* 2015) have also been reported. It is highly likely that co-variation of traits relevant to seed dispersal is as extensive with wind dispersal as with frugivory.

Complexity also arises in animal-dispersed species because foraging animals often make foraging decisions hierarchically (Côrtes and Uriarte 2013). For example, foraging frugivores must first select the foraging patch, then choose the individuals to feed in, and then choose which fruits to harvest from that plant. In addition, multiple cues may be used hierarchically at any single stage of this process. For example, experiments with the large fleshy-fruited shrub *C. monogyna* elegantly demonstrated hierarchical selection by *Turdus migratorius* of individual trees in which to forage. First, birds preferred trees with larger crop sizes, but if crop sizes were constant, they preferred plants with larger fruits, and, finally, if fruit size was constant, they preferred plants with greater pulp:seed ratios (Sallabanks 1993).

Understanding variation in seed dispersal is further complicated by the concomitant interindividual variation in seed dispersers, including sexual dimorphism, ontogenetic changes, interindividual variation in specialization and unique animal personalities (Zwolak 2018). For example, our discussion of fruit size variation in *M. communis* and its effect on fruit availability to different seed dispersers was based on measured intraindividual and interindividual variation in fruit diameters but only mean gape width for the dispersal agents (González-Varo and Traveset 2016). Interpretations could be different if interindividual variation in the seed disperser species was also incorporated. More generally, interindividual variation in plants and dispersers interact and it might be difficult to understand one without understanding the other (Côrtes and Uriarte 2013). Plants almost certainly respond at the individual level to variation in how seed dispersers interact with them; these eco-evolutionary feedbacks mean that intraspecific variation is important in both sides of the interaction, perhaps even intensifying the individual-level variation in both players (compare with Siepielski and Benkman 2010).

Further complexity is likely in particular dispersal systems, such as for example with diplochorous dispersal, where dispersal is accomplished by a sequence of steps that involve different dispersal agents such as primary dispersal by a frugivorous bird and secondary dispersal by a rodent (Vander Wall and Longland 2004). We predict that, all else being equal, diplochorous dispersal systems would have even greater interindividual variation in seed dispersal success than non-diplochorous systems given that variability arising during the second phase of dispersal is building on variability created during the first phase of dispersal. For example, as discussed previously, intraspecific variation in seed size can affect selection by both frugivorous birds and rodents, sometimes in the same way and sometimes not.

Lastly, all of these trait-based dispersal drivers are playing out in an extraordinarily heterogeneous environment, varying continuously in habitat structure, fruiting neighbourhoods, wind conditions and more.

## Where Are We Now and Where Do We Need to Go?

While we show substantial evidence that drivers of intraspecific variation in seed dispersal are diverse and pervasive, we also reveal large gaps in our understanding, partly due to a paucity of research directly addressing intraspecific, especially interindividual, variation in seed dispersal, and partly due to the complexity of interactions among drivers. Our understanding is limited further by the existing empirical work’s focus on the quantity of seed dispersal, with much less consideration of the quality of dispersal or LDD. Of particular interest are the intrinsic trait-based drivers that can respond to natural selection. The best-supported and best-understood intrinsic driver of interindividual variation in seed dispersal is crop size; with more seeds produced, more seeds are dispersed. Crop size is also likely the most widespread driver, being relevant to most if not all forms of dispersal. Though less well supported and less well understood, fruit/seed size is likely the second most widespread intrinsic driver. Again, it seems to be relevant to a broad range of seed dispersal modes. However, when it comes to animal-mediated dispersal we do not have a good understanding of the ultimate cause of size-based fruit or seed selection—is it fruit/seed size *per se*, or some co-varying trait such as pulp:seed ratio? Remaining intrinsic drivers are even more poorly understood, though apparently range from widespread but weak, such as plant height, to sporadic and variable in strength, such as colour polymorphism. For extrinsic drivers, a variety of studies have addressed the impact of fruiting neighbourhoods on interindividual variation in seed dispersal, but we do not understand well when to expect competition for dispersers and when to expect facilitation of dispersal. With respect to habitat structure, much relevant work has been from the perspective of anthropogenic impacts of habitat fragmentation and degradation on seed dispersal rather than from the perspective of interspecific variation in seed dispersal.

Beyond limited empirical work, we are further hindered by an even greater lack of theory related to the drivers of intraspecific, especially interindividual variation in seed dispersal. While there have been some theoretical developments around fruit crop size and seed dispersal success (see earlier discussion of Howe and Estabrook 1977; Carlo *et al.* 2007), we are aware of no other developed theory that can guide our understanding of the drivers of interindividual variation in dispersal and potential demographic and evolutionary responses to such variation.

Looking forward towards potential research directions, in Box 2 we highlight a selection of outstanding questions concerning intrinsic drivers of intraspecific variation in seed dispersal that we personally believe to be especially informative and intriguing to answer. We present these questions as a starting point to advance our understanding of intraspecific drivers of seed dispersal. One promising approach to answer these questions and disentangle the complexity inherent in intraspecific seed dispersal is a frugivore-centred modelling approach (Côrtes and Uriarte 2013). This approach advocates parameterizing field data on intrinsic animal factors and behaviour, as well as extrinsic landscape factors, to test and quantify the strength of the variables affecting the spatially explicit deposition of seeds across the landscape (Côrtes and Uriarte 2013). Mechanistic simulations can be used in a hierarchical manner to test the effect of multiple factors one at a time, to quantify their relative influence on patterns of seed deposition (Côrtes and Uriarte 2013). Studies using this approach have successfully quantified the impact on seed dispersal of edge-following behaviour in a fragmented landscape (Levey *et al.* 2005), fruiting neighbourhoods (Carlo and Morales 2008) and drivers of reduced LDD (Uriarte *et al.* 2011). Although primarily envisioned to study endozoochory, similar methods have been applied to epizoochory (Will and Tackenberg 2008) and other dispersal modes by considering relevant intrinsic and extrinsic factors (e.g. anemochory, Nathan *et al.* 2001). Additionally, a powerful molecular approach that matches individual seeds or seedlings to maternal plants (Grivet *et al.* 2009) across dispersal modes is also promising for studying individual variation in seed dispersal and may compliment simulation modelling approaches. Despite the daunting complexity of drivers of intraspecific variation in seed dispersal, the combination of quantitative approaches and tools available provide ample starting points to answer the questions we pose in Box 2 and improve our understanding of this important aspect of seed dispersal.

Box 1. **The Consequences of Seed Dispersal: Seed Dispersal Effectiveness and Long Distance Dispersal**

Seed dispersal effectiveness, or SDE, can be defined ideally as the contribution a seed disperser makes to the production of new reproductive adults of a plant it disperses, whether the ‘disperser’ is a frugivorous bird, a seed-caching rodent or the wind (Schupp 1993; Schupp *et al.* 2010, 2017). SDE = quantity × quality, where quantity is the number of seeds dispersed and quality is the probability that a dispersed seed successfully produces a new adult. However, in practical terms empirical studies are generally restricted to quantifying the contribution to some earlier relevant stage such as seedling establishment rather than new adults.

From the perspective of this review, the quantity of seed dispersal is straightforward and well-studied: the number of seeds dispersed. Quality, on the other hand, is influenced by a number of attributes of dispersal that arise repeatedly in this review. Three particularly important and frequent attributes of dispersal that arise when considering intraspecific variation in seed dispersal are briefly highlighted below.

**Distance dispersed:** The distance seeds are dispersed from the parent can affect the quality of dispersal in several ways. The most widely recognized consequence is increased survival by escaping from distance- and density-dependent seed and seedling enemies that concentrate attack beneath and near adult conspecifics (e.g. Janzen 1970; Connell 1971; Howe *et al.* 1985; Schupp 1988; Comita *et al.* 2014). Longer dispersal distances may also increase the chances of reaching unpredictably located suitable sites (Norghauer *et al.* 2011). Lastly, longer distance dispersal is important for gene flow and colonization of new sites (Nathan 2006; García *et al.* 2007; Jordano 2017). This attribute of dispersal is applicable to all modes of dispersal, biotic and abiotic.

**Dispersal destination:** Where in the landscape a seed is deposited can be described by the biotic and abiotic environments the potential recruit faces (Schupp and Fuentes 1995)—the seedscape (Beckman and Rogers 2013)—and these environments interact with the seed/seedling to determine its fate. There is substantial evidence that the habitat or microhabitat in which a seed is deposited, whether by a bird defecating, a rodent caching or a floating seed landing, has a large influence on seed and seedling fate (e.g. Schupp 2007; Zhang *et al.* 2013; Young and Kelly 2018). Further, whether seeds are deposited widely scattered or in high density clumps at latrines, sleeping trees or favourite processing sites influences seedling competition and susceptibility to density-dependent natural enemies independent of distance from the parent (e.g. Schupp *et al.* 2002).

**Treatment in the mouth and gut:** For animal-vectored dispersal, the first critical distinction is whether all seeds are being treated gently and dispersed physically intact or whether some-to-many are broken or damaged (Schupp 1993). Secondly, for intact seeds it can matter whether the seed is (i) dropped after some of the pulp has been picked off and consumed, (ii) swallowed and either regurgitated or spit out clean or (iii) swallowed, passed through the digestive track, and defecated. These different pathways can result in differences in germination (e.g. Rodríguez-Pérez *et al.* 2005; Reid and Armesto 2011; Haurez *et al.* 2018) and in post-dispersal interactions with seed predators and secondary dispersers (e.g. Fricke *et al.* 2016; Pan *et al.* 2016; Guerra *et al.* 2018).

Box 2. **Questions**

There is an abundance of questions that remain to be answered. Here we highlight a selection of outstanding questions concerning intrinsic drivers of intraspecific variation in seed dispersal that we personally believe to be especially informative and intriguing to answer.

**General**

• How strong and widespread are the major drivers of intraspecific variation in seed dispersal? Do their relative strengths differ across dispersal modes, and to what extent do drivers operate independently versus interactively?
• What is the relative contribution of intraindividual versus interindividual variation in traits to variation in seed dispersal patterns and SDE?
• How variable is the extent of intraindividual variation in dispersal traits and dispersal patterns, both within and among populations? Do the answers to these questions depend on dispersal mode?

**Plant–Animal Dispersal Mutualisms**

• When and to what extent do animal seed dispersers respond to intraindividual versus interindividual variation in fruit or seed traits? To what extent do seed dispersers respond to interindividual mean versus variance in fruit or seed traits?
• How does interindividual variation in plant traits interact with interindividual variation in seed disperser traits to affect patterns of seed dispersal and SDE?

## References

[CIT0001] Albert A, Auffret AG, Cosyns E, Cousins SAO, D’hondt B, Eichberg C, Eycott AE, Heinken T, Hoffmann M, Jaroszewicz B, Malo JE, Mårell A, Mouissie M, Pakeman RJ, Picard M, Plue J, Poschlod P, Provoost S, Schulzem KA, Baltzinger C 2015 Seed dispersal by ungulates as an ecological filter: a trait-based meta-analysis. Oikos 124:1109–1120.

[CIT0002] Albrecht J, Bohle V, Berens DG, Jaroszewicz B, Selva N, Farwig N 2015 Variation in neighbourhood context shapes frugivore-mediated facilitation and competition among co-dispersed plant species. Journal of Ecology 103:526–536.

[CIT0003] Albrecht J, Hagge J, Schabo DG, Schaefer HM, Farwig N 2018 Reward regulation in plant-frugivore networks requires only weak cues. Nature Communications 9:4838. 10.1038/s41467-018-07362-z PMC6240120 30446651

[CIT0004] Alcántara JM, Rey PJ, Valera F, Sainchez-Lafuente AM, Gutiérrez JE 1997 Habitat alteration and plant intra-specific competition for seed dispersers. An example with *Olea europaea* var. *sylvestris.* Oikos 79:291–300.

[CIT0005] Alves BC, Mendes CP, Ribeiro MC 2018 Queen palm fruit selection and foraging techniques of squirrels in the Atlantic Forest. Biotropica 50:274–281.

[CIT0006] Augspurger CK, Franson SE, Cushman KC 2017 Wind dispersal is predicted by tree, not diaspore, traits in comparisons of Neotropical species. Functional Ecology 31:808–820.

[CIT0007] Baldwin JW, Whitehead SR 2015 Fruit secondary compounds mediate the retention time of seeds in the guts of Neotropical fruit bats. Oecologia 177:453–466. 25262120 10.1007/s00442-014-3096-2

[CIT0008] Baskin CC, Baskin JM 2014 Seeds, ecology, biogeography, and evolution of dormancy and germination, 2nd edn. San Diego, CA: Academic Press.

[CIT0009] Battaglia LL, Denslow JS, Inczauskis JR, Baer SG 2009 Effects of native vegetation on invasion success of Chinese tallow in a floating marsh ecosystem. Journal of Ecology 97:239–246.

[CIT0010] Beckman NG, Rogers HS 2013 Consequences of seed dispersal for plant recruitment in tropical forests: interactions within the seedscape. Biotropica 45:666–681.

[CIT0011] Berg H 2000 Differential seed dispersal in *Oxalis acetosella*, a cleistogamous perennial herb. Acta Oecologica 21:109–118.

[CIT0012] Bhuyan P, Khan ML, Shankar U 2000 Trade-off between dispersal efficiency and seedling fitness in *Oroxylum indicum*, a wind-dispersed tropical tree. International Journal of Ecology and Environmental Sciences 26:67–73.

[CIT0013] Blendinger PG, Loiselle BA, Blake JG 2008 Crop size, plant aggregation, and microhabitat type affect fruit removal by birds from individual melastome plants in the Upper Amazon. Oecologia 158:273–283. 18810498 10.1007/s00442-008-1146-3

[CIT0014] Blendinger PG, Villegas M 2011 Crop size is more important than neighborhood fruit availability for fruit removal of *Eugenia uniflora* (Myrtaceae) by bird seed dispersers. Plant Ecology 212:889–899.

[CIT0015] Bogdziewicz M, Crone EE, Steele MA, Zwolak R 2017 Effects of nitrogen deposition on reproduction in a masting tree: benefits of higher seed production are trumped by negative biotic interactions. Journal of Ecology 105:310–320.

[CIT0016] Bogdziewicz M, Lichti NI, Zwolak R 2019 Consumer‐mediated indirect interaction with a native plant lowers the fitness of an invasive competitor. Journal of Ecology 107:12–22 .

[CIT0017] Bogdziewicz M, Marino S, Bonal R, Zwolak R, Steele MA 2018 Rapid aggregative and reproductive responses of weevils to masting of North American oaks counteract predator satiation. Ecology 99:2575–2582. 30182480 10.1002/ecy.2510

[CIT0018] Bohrer G, Katul GG, Nathan R, Walko RL, Avissar R 2008 Effects of canopy heterogeneity, seed abscission and inertia on wind-driven dispersal kernels of tree seeds. Journal of Ecology 96:569–580.

[CIT0019] Boieiroa M, Espadaler X, Gómez C, Eustaquio A 2012 Spatial variation in the fatty acid composition of elaiosomes in an ant-dispersed plant: differences within and between individuals and populations. Flora 207:497–502.

[CIT0020] Bolnick DI, Amarasekare P, Araújo MS, Bürger R, Levine JM, Novak M, Rudolf VH, Schreiber SJ, Urban MC, Vasseur DA 2011 Why intraspecific trait variation matters in community ecology. Trends in Ecology & Evolution 26:183–192. 21367482 10.1016/j.tree.2011.01.009 PMC3088364

[CIT0021] Bossema I 1979 Jays and oaks: an eco-ethological study of a symbiosis. Behaviour 70:1–117.

[CIT0022] Boulay R, Coll-Toledano J, Manzaneda AJ, Cerdá X 2007 Geographic variations in seed dispersal by ants: are plant and seed traits decisive? Die Naturwissenschaften 94:242–246. 17119907 10.1007/s00114-006-0185-z

[CIT0023] Bravo S 2012 From which *Ocotea diospyrifolia* trees does *Alouatta caraya* (Primates, Atelidae) eat fruits? Journal of Tropical Ecology 28:417–420.

[CIT0024] Breviglieri CP, Piccoli GC, Uieda W, Romero GQ 2013 Predation-risk effects of predator identity on the foraging behaviors of frugivorous bats. Oecologia 173:905–912. 23657559 10.1007/s00442-013-2677-9

[CIT0025] Breviglieri CPB, Romero GQ 2016 Snakes and forbidden fruits: non-consumptive effects of snakes on the behaviors of frugivorous birds. Behavioral Ecology and Sociobiology 70:777–783.

[CIT0026] Brewer SW 2001 Predation and dispersal of large and small seeds of a tropical palm. Oikos 92:245–255.

[CIT0027] Bullock JM, Moy IL, Coulson SJ, Clarke RT 2003 Habitat-specific dispersal: environmental effects on the mechanisms and patterns of seed movement in a grassland herb *Rhinanthus minor*. Ecography 26:692–704.

[CIT0028] Calviño-Cancela M, Martín-Herrero J 2009 Effectiveness of a varied assemblage of seed dispersers of a fleshy-fruited plant. Ecology 90:3503–3515. 20120817 10.1890/08-1629.1

[CIT0029] Cao L, Wang Z, Yan C, Chen J, Guo C, Zhang Z 2016 Differential foraging preferences on seed size by rodents result in higher dispersal success of medium-sized seeds. Ecology 97:3070–3078. 27870042 10.1002/ecy.1555

[CIT0030] Carlo TA 2005 Interspecific neighbors change seed dispersal pattern of an avian‐dispersed plant. Ecology 86:2440–2449.

[CIT0031] Carlo TA, Aukema JE, Morales JM 2007 Plant-frugivore interactions as spatially explicit networks: integrating frugivore foraging with fruiting plant spatial patterns. In: Dennis AJ, Schupp EW, Green RJ, Westcott DA, eds. Seed dispersal. Theory and its application in a changing world. Wallingford, UK: CAB International, 369–390.

[CIT0032] Carlo TA, Morales JM 2008 Inequalities in fruit‐removal and seed dispersal: consequences of bird behaviour, neighbourhood density and landscape aggregation. Journal of Ecology 96:609–618.

[CIT0033] Cazetta E, Galetti M, Rezende EL, Schaefer HM 2012 On the reliability of visual communication in vertebrate-dispersed fruits. Journal of Ecology 100:277–286.

[CIT0034] Cazetta E, Schaefer HM, Galetti M 2008 Does attraction to frugivores or defense against pathogens shape fruit pulp composition? Oecologia 155:277–286. 18043946 10.1007/s00442-007-0917-6

[CIT0035] Chatterjee S, Basu P 2015 Avian frugivory and seed dispersal of a large fruited tree in an Indian moist deciduous forest. Acta Oecologica 65–66:32–40.

[CIT0036] Chen J, Deng X-B, Bai Z-L, Yang Q, Chen G-Q, Liu Y, Liu Z-Q 2001 Fruit characteristics and *Munfiacus muntijak vaginalis* (Muntjac) visits to individual plants of *Choerospondias axillaris*. Biotropica 33:718–722.

[CIT0037] Cheng J, Zhang H 2011 Seed-hoarding of Edward’s long-tailed rats *Leopoldamys edwardsi* in response to weevil infestation in cork oak *Quercus variabilis*. Current Zoology 57:50–55.

[CIT0038] Clark J, Fastie C, Hurtt G, Jackson S, Johnson C, King G, Lewis M, Lynch J, Pacala S, Prentice C, Schupp EW, Webb T III, Wyckoff P 1998 Dispersal theory offers solutions to Reid’s paradox of rapid plant migration. BioScience 48:13–24.

[CIT0039] Collier MH, Rogstad SH 2004 Clonal variation in floral stage timing in the common dandelion *Taraxacum officinale* (Asteraceae). American Journal of Botany 91:1828–1833. 21652330 10.3732/ajb.91.11.1828

[CIT0040] Comita LS, Queenborough SA, Murphy SJ, Eck JL, Xu K, Krishnadas M, Beckman N, Zhu Y, Gómez-Aparicio L 2014 Testing predictions of the Janzen-Connell hypothesis: a meta-analysis of experimental evidence for distance- and density-dependent seed and seedling survival. The Journal of Ecology 102:845–856. 25253908 10.1111/1365-2745.12232 PMC4140603

[CIT0041] Connell JH 1971 On the role of natural enemies in preventing competitive exclusion in some marine animals and in rain forest trees. In: Den Boer PJ, Gradwell GR, eds. Dynamics of populations. Wageningen, The Netherlands: PUDOC, 298–312.

[CIT0042] Coopman RE, Reyes-Díaz M, Briceño VF, Corcuera LJ, Cabrera HM, Bravo LA 2008 Changes during early development in photosynthetic light acclimation capacity explain the shade to sun transition in *Nothofagus nitida*. Tree Physiology 28:1561–1571. 18708338 10.1093/treephys/28.10.1561

[CIT0043] Cordeiro NJ, Howe HF 2003 Forest fragmentation severs mutualism between seed dispersers and an endemic African tree. Proceedings of the National Academy of Sciences of the United States of America 100:14052–14056. 14614145 10.1073/pnas.2331023100 PMC283544

[CIT0044] Corlett RT, Lucas PW 1990 Alternative seed-handling strategies in primates: seed-spitting by long-tailed macaques (*Macaca fascicularis*). Oecologia 82:166–171. 28312661 10.1007/BF00323531

[CIT0045] Côrtes MC, Uriarte M 2013 Integrating frugivory and animal movement: a review of the evidence and implications for scaling seed dispersal. Biological Reviews of the Cambridge Philosophical Society 88:255–272. 23136896 10.1111/j.1469-185X.2012.00250.x

[CIT0046] Cramer JM, Mesquita RC, Bentos TV, Moser B, Williamson GB 2007 Forest fragmentation reduces seed dispersal of *Duckeodendron cestroides*, a Central Amazon endemic. Biotropica 39:709–718.

[CIT0047] Crestani AC, Mello MAR, Cazetta E 2019 Interindividual variations in plant and fruit traits affect the structure of a plant-frugivore network. Acta Oecologica 95:120–127.

[CIT0048] Davi H, Cailleret M, Restoux G, Amm A, Pichot C, Fady B 2016 Disentangling the factors driving tree reproduction. Ecosphere 7:e01389.

[CIT0049] Davies KW, Sheley RL 2007 Influence of neighboring vegetation height on seed dispersal: implications for invasive plant management. Weed Science 55:626–630

[CIT0050] Debain S, Curt T, Lepart J 2003 Seed mass, seed dispersal capacity, and seedling performance in a *Pinus sylvestris* population. Écoscience 10:168–175.

[CIT0051] De Clavijo ER 2005 The reproductive strategies of the heterocarpic annual *Calendula arvensis* (Asteraceae). Acta Oecologica 28:119–126.

[CIT0052] Delefosse M, Povidisa K, Poncet D, Kristensen E, Olesen B 2016 Variation in size and chemical composition of seeds from the seagrass *Zostera marina*—ecological implications. Aquatic Botany 131:7–14.

[CIT0053] Denslow JS 1987 Fruit removal rates from aggregated and isolated bushes of the red elderberry, *Sambucus pubens*. Canadian Journal of Botany 65:1229–1235.

[CIT0054] De Rouw A, Ribolzi O, Douillet M, Tjantahosong H, Soulileuth B 2018 Weed seed dispersal via runoff water and eroded soil. Agriculture, Ecosystems & Environment 265:488–502.

[CIT0055] DiTommaso A, Stokes CA, Cordeau S, Milbrath LR, Whitlow TH 2018 Seed-dispersal ability of the invasive perennial vines *Vincetoxicum nigrum* and *Vincetoxicum rossicum*. Invasive Plant Science and Management 11:10–19.

[CIT0056] Donohue K 1998 Maternal determinants of seed dispersal in *Cakile edentula*: fruit, plant, and site traits. Ecology 79:2771–2788.

[CIT0057] Effiom EO, Nuñez-Iturri G, Smith HG, Ottosson U, Olsson O 2013 Bushmeat hunting changes regeneration of African rainforests. Proceedings of the Royal Society of London B. Biological Sciences 280:20130246. 10.1098/rspb.2013.0246 PMC3619513 23516245

[CIT0058] Espelta JM, Cortés P, Molowny-Horas R, Sánchez-Humanes B, Retana J 2008 Masting mediated by summer drought reduces acorn predation in Mediterranean oak forests. Ecology 89:805–817. 18459343 10.1890/07-0217.1

[CIT0059] Farwig N, Schabo DG, Albrecht J 2017 Trait-associated loss of frugivores in fragmented forest does not affect seed removal rates. Journal of Ecology 105:20–28.

[CIT0060] Fischer SF, Poschlod P, Beinlich B 1996 Experimental studies on the dispersal of plants and animals on sheep in calcareous grasslands. Journal of Applied Ecology 33:1206–1222.

[CIT0061] Fischer RC, Richter A, Hadacek F, Mayer V 2008 Chemical differences between seeds and elaiosomes indicate an adaptation to nutritional needs of ants. Oecologia 155:539–547. 18095003 10.1007/s00442-007-0931-8

[CIT0062] Flörchinger M, Braun J, Böhning-Gaese K, Schaefer HM 2010 Fruit size, crop mass, and plant height explain differential fruit choice of primates and birds. Oecologia 164:151–161. 20490552 10.1007/s00442-010-1655-8

[CIT0063] Fontúrbel FE, Candia AB, Malebrán J, Salazar DA, González-Browne C, Medel R 2015 Meta-analysis of anthropogenic habitat disturbance effects on animal-mediated seed dispersal. Global Change Biology 21:3951–3960. 26149368 10.1111/gcb.13025

[CIT0064] Franklin DC, Bach CS 2006 Assessing intraspecific phenological synchrony in zoochorous trees from the monsoon forests of northern Australia. Journal of Tropical Ecology 22:419–429.

[CIT0065] Fricke EC, Haak DC, Levey DJ, Tewksbury JJ 2016 Gut passage and secondary metabolites alter the source of post-dispersal predation for bird-dispersed chili seeds. Oecologia 181:905–910. 27016078 10.1007/s00442-016-3612-7

[CIT0066] Fuentes M, Guitián J, Guitián P, Bermejo T, Larrinaga A, Amézquita P, Bongiomo S 2001 Small-scale spatial variation in the interactions between *Prunus mahaleb* and fruit-eating birds. Plant Ecology 157:69–75.

[CIT0067] Fukano Y, Hirayama H, Tanaka K 2014 A herbivory-induced increase in the proportion of floating seeds in an invasive plant. Acta Oecologica 56:27–31.

[CIT0068] Galetti M, Guevara R, Côrtes MC, Fadini R, Von Matter S, Leite AB, Labecca F, Ribeiro T, Carvalho CS, Collevatti RG, Pires MM, Guimarães PR Jr, Brancalion PH, Ribeiro MC, Jordano P 2013 Functional extinction of birds drives rapid evolutionary changes in seed size. Science 340:1086–1090. 23723235 10.1126/science.1233774

[CIT0069] Gálvez D, Jansen PA 2007 Bruchid beetle infestation and the value of *Attalea butyracea* endocarps for neotropical rodents. Journal of Tropical Ecology, 23:381–384.

[CIT0070] García C, Jordano P, Godoy JA 2007 Contemporary pollen and seed dispersal in a *Prunus mahaleb* population: patterns in distance and direction. Molecular Ecology 16:1947–1955. 17444903 10.1111/j.1365-294X.2006.03126.x

[CIT0071] García D, Zamora R, Gómez JM, Hódar JA 2001 Frugivory at *Juniperus communis* depends more on population characteristics than on individual attributes. Journal of Ecology 89:639–647.

[CIT0072] Gervais JA, Noon BR, Willson MF 1999 Avian selection of the color-dimorphic fruits of salmonberry, *Rubus spectabilis*: a field experiment. Oikos 84:77–86.

[CIT0073] Gómez JM 2007 Dispersal-mediated selection on plant height in an autochorously dispersed herb. Plant Systematics and Evolution 268:119–130.

[CIT0074] Gómez JM, Puerta-Piñero C, Schupp EW 2008 Effectiveness of rodents as local seed dispersers of Holm oaks. Oecologia 155:529–537. 18075760 10.1007/s00442-007-0928-3

[CIT0075] Gómez JM, Schupp EW, Jordano P 2019 Synzoochory: the ecological and evolutionary relevance of a dual interaction. Biological Reviews of the Cambridge Philosophical Society 94:874–902. 30467946 10.1111/brv.12481

[CIT0076] González-Varo JP, Arroyo JM, Jordano P 2019 The timing of frugivore-mediated seed dispersal effectiveness. Molecular Ecology 28:219–231. 30151871 10.1111/mec.14850 PMC6905405

[CIT0077] González-Varo JP, Carvalho CS, Arroyo JM, Jordano P 2017 Unravelling seed dispersal through fragmented landscapes: frugivore species operate unevenly as mobile links. Molecular Ecology 26:4309–4321. 28503829 10.1111/mec.14181

[CIT0078] González-Varo JP, Traveset A 2016 The labile limits of forbidden interactions. Trends in Ecology & Evolution 31:700–710. 27471077 10.1016/j.tree.2016.06.009

[CIT0079] Gorb E, Gorb S 2002 Contact separation force of the fruit burrs in four plant species adapted to dispersal by mechanical interlocking. Plant Physiology and Biochemistry 40:373–381.

[CIT0080] Graae BJ 2002 The role of epizoochorous seed dispersal of forest plant species in a fragmented landscape. Seed Science Research 12:113–121.

[CIT0081] Gravuer K, von Wettberg EJ, Schmitt J 2003 Dispersal biology of *Liatris scariosa* var. *novae-angliae* (Asteraceae), a rare New England grassland perennial. American Journal of Botany 90:1159–1167. 21659216 10.3732/ajb.90.8.1159

[CIT0082] Grivet D, Robledo-Arnuncio JJ, Smouse PE, Sork VL 2009 Relative contribution of contemporary pollen and seed dispersal to the effective parental size of seedling population of California valley oak (*Quercus lobata*, Née). Molecular Ecology 18:3967–3979. 19754515 10.1111/j.1365-294X.2009.04326.x

[CIT0083] Gryj EO, Domínguez CA 1996 Fruit removal and postdispersal survivorship in the tropical dry forest shrub *Erythroxylum havanense*: ecological and evolutionary implications. Oecologia 108:368–374. 28307851 10.1007/BF00334663

[CIT0084] Guerra TJ, Dayrell RLC, Arruda AJ, Dáttilo W, Teixido AL, Messeder JVS, Silveira FAO 2017 Intraspecific variation in fruit-frugivore interactions: effects of fruiting neighborhood and consequences for seed dispersal. Oecologia 185:233–243. 28875387 10.1007/s00442-017-3943-z

[CIT0085] Guerra TJ, Messeder JVS, Arruda AJ, Fuzessy LF, Dayrell RLC, Neves FS, Silveira FAO 2018 Handling by avian frugivores affects diaspore secondary removal. PLoS One 13:e0202435. 30157261 10.1371/journal.pone.0202435 PMC6114891

[CIT0086] Guitián J, Fuentes M, Bermejo T, López B 1992 Spatial variation in the interactions between *Prunus mahaleb* and frugivorous birds. Oikos 63:125–130.

[CIT0087] Guitián J, Munilla I 2010 Responses of mammal dispersers to fruit availability: rowan (*Sorbus aucuparia*) and carnivores in mountain habitats of northern Spain. Acta Oecologica 36:242–247.

[CIT0088] Guja LK, Merritt DJ, Dixon KW, Wardell-Johnson G 2014 Dispersal potential of *Scaevola crassifolia* (Goodeniaceae) is influenced by intraspecific variation in fruit morphology along a latitudinal environmental gradient. Australian Journal of Botany 62:56–64.

[CIT0089] Hampe A 2008 Fruit tracking, frugivore satiation, and their consequences for seed dispersal. Oecologia 156:137–145. 18270742 10.1007/s00442-008-0979-0

[CIT0090] Haurez B, Tagg N, Petre C-A, Brostaux Y, Boubady A, Doucet J-L 2018 Seed dispersal effectiveness of the western lowland gorilla (*Gorilla gorilla gorilla*) in Gabon. African Journal of Ecology 56:185–193.

[CIT0091] Hernández Á 2009 Birds and guelder rose *Viburnum opulus*: selective consumption and dispersal via regurgitation of small-sized fruits and seeds. Plant Ecology 203:111–122.

[CIT0092] Herrera CM 2009 Multiplicity in unity. Plant subindividual variation and interactions with animals. Chicago, IL: University of Chicago Press.

[CIT0093] Herrera CM 2017 The ecology of subindividual variability in plants: patterns, processes, and prospects. Web Ecology 17:51–64.

[CIT0094] Herrera CM, Jordano P, López-Soria L, Amat JA 1994 Recruitment of a mast-fruiting, bird-dispersed tree: bridging frugivore activity and seedling establishment. Ecological Monographs 64:315–344.

[CIT0095] Herrera JM, Morales JM, García D 2011 Differential effects of fruit availability and habitat cover for frugivore-mediated seed dispersal in a heterogeneous landscape. Journal of Ecology 99:1100–1107.

[CIT0096] Herrera JM, de Sá Teixeira I, Rodríguez-Pérez J, Mira A 2016 Landscape structure shapes carnivore-mediated seed dispersal kernels. Landscape Ecology 31:731–743.

[CIT0097] Houle A, Chapman CA, Vickery WL 2007 Intratree variation in fruit production and implications for primate foraging. International Journal of Primatology 28:1197–1217.

[CIT0098] Hovstad KA, Borvik S, Ohlson M 2009 Epizoochorous seed dispersal in relation to seed availability–an experiment with a red fox dummy. Journal of Vegetation Science 20:455–464.

[CIT0099] Howe HF, De Steven D 1979 Fruit production, migrant bird visitation, and seed dispersal of *Guarea glabra* in Panama. Oecologia 39:185–196. 28309435 10.1007/BF00348067

[CIT0100] Howe HF, Estabrook GF 1977 On intraspecific competition for avian dispersers in tropical trees. American Naturalist 111:817–832.

[CIT0101] Howe HF, Schupp EW, Westley LC 1985 Early consequences of seed dispersal for a neotropical tree (*Virola surinamensis*). Ecology 66:781–791.

[CIT0102] Howe HF, Vande Kerckhove GA 1979 Fecundity and seed dispersal of a tropical tree. Ecology 60:180–189.

[CIT0103] Howe HF, Vande Kerckhove GA 1981 Removal of wild nutmeg (*Virola surinamensis*) crops by birds. Ecology 62:1093–1106.

[CIT0104] Hughes L, Dunlop M, French K, Leishman MR, Rice B, Rodgerson L, Westoby M 1994 Predicting dispersal spectra: a minimal set of hypotheses based on plant attributes. Journal of Ecology 82:933–950.

[CIT0105] Iida S 2004 Indirect negative influence of dwarf bamboo on survival of *Quercus* acorn by hoarding behavior of wood mice. Forest Ecology and Management 202:257–263.

[CIT0106] Imbert E, Ronce O 2001 Phenotypic plasticity for dispersal ability in the seed heteromorphic *Crepis sancta* (Asteraceae). Oikos 93:126–134.

[CIT0107] Izhaki I, Tsahar E, Paluy I, Friedman J 2002 Within population variation and interrelationships between morphology, nutritional content, and secondary compounds of *Rhamnus alaternus* fruits. New Phytologist 156:217–223. 10.1046/j.1469-8137.2002.00515.x 33873272

[CIT0108] Jacobs BS, Lesmeister SA 2012 Maternal environmental effects on fitness, fruit morphology and ballistic dispersal distance in an annual forb. Functional Ecology 26:588–597.

[CIT0109] Jansen PA, Bongers F, Hemerik L 2004 Seed mass and mast seeding enhance dispersal by a neotropical scatter-hoarding rodent. Ecological Monographs 74:569–589.

[CIT0110] Jansen PA, Visser MD, Joseph Wright S, Rutten G, Muller-Landau HC 2014 Negative density dependence of seed dispersal and seedling recruitment in a neotropical palm. Ecology Letters 17:1111–1120. 25039608 10.1111/ele.12317

[CIT0111] Janzen DH 1970 Herbivores and the number of tree species in tropical forests. American Naturalist 104:501–529.

[CIT0112] Jones LR, Duke-Sylvester SM, Leberg PL, Johnson DM 2017 Closing the gaps for animal seed dispersal: separating the effects of habitat loss on dispersal distances and seed aggregation. Ecology and Evolution 7:5410–5425. 28770078 10.1002/ece3.3113 PMC5528214

[CIT0113] Jordano P 1984 Seed weight variation and differential avian dispersal in blackberries *Rubus ulmifolius*. Oikos 43:149–153.

[CIT0114] Jordano P 1987 Avian fruit removal: effects of fruit variation, crop size, and insect damage. Ecology 68:1711–1723. 29357140 10.2307/1939863

[CIT0115] Jordano P 1992 Fruit and frugivory. In: Fenner M, ed. Seeds: the ecology of regeneration in plant communities. Wallingford, UK: CAB International, 105–151.

[CIT0116] Jordano P 1995 Frugivore-mediated selection on fruit and seed size: birds and St. Lucie’s cherry, *Prunus mahaleb*. Ecology 76:2627–2639.

[CIT0117] Jordano P 2017 What is long-distance dispersal? And a taxonomy of dispersal events. Journal of Ecology 105:75–84.

[CIT0118] Jordano P, Schupp EW 2000 Seed disperser effectiveness: the quantity component and patterns of seed rain for *Prunus mahaleb*. Ecological Monographs 70:591–615.

[CIT0119] Jorge ML, Howe HF 2009 Can forest fragmentation disrupt a conditional mutualism? A case from central Amazon. Oecologia 161:709–718. 19633870 10.1007/s00442-009-1417-7

[CIT0120] Kellner KF, Lichti NI, Swihart RK 2016 Midstory removal reduces effectiveness of oak (*Quercus*) acorn dispersal by small mammals in the Central Hardwood Forest region. Forest Ecology and Management 375:182–190.

[CIT0121] Kunz BK, Linsenmair KE 2008 Seed size selection by olive baboons. Primates 49:239–245. 18780144 10.1007/s10329-008-0101-6

[CIT0122] Kuprewicz EK, García-Robledo C 2019 Deciphering seed dispersal decisions: size, not tannin content, drives seed fate and survival in a tropical forest. Ecosphere 10:e02551.

[CIT0123] Lahann P 2007 Feeding ecology and seed dispersal of sympatric cheirogaleid lemurs (*Microcebus murinus*, *Cheirogaleus medius*, *Cheirogaleus major*) in the littoral rainforest of south-east Madagascar. Journal of Zoology 271:88–98.

[CIT0124] Larios E, Venable DL 2015 Maternal adjustment of offspring provisioning and the consequences for dispersal. Ecology 96:2771–2780. 26649397 10.1890/14-1565.1

[CIT0125] Larrinaga AR 2010 Rabbits (*Oryctolagus cuniculus*) select small seeds when feeding on the fruits of *Corema album*. Ecological Research 25:245–249.

[CIT0126] Lehouck V, Spanhove T, Colson L, Adringa‐Davis A, Cordeiro NJ, Lens L 2009 Habitat disturbance reduces seed dispersal of a forest interior tree in a fragmented African cloud forest. Oikos 118:1023–1034.

[CIT0127] Levey DJ 1987 Sugar-tasting ability and fruit selection in tropical fruit-eating birds. The Auk 104:173–179.

[CIT0128] Levey DJ, Bolker BM, Tewksbury JJ, Sargent S, Haddad NM 2005 Effects of landscape corridors on seed dispersal by birds. Science 309:146–148. 15994561 10.1126/science.1111479

[CIT0129] Levey DJ, Cipollini ML 1998 A glycoalkaloid in ripe fruit deters consumption by cedar waxwings. The Auk 115:359–367.

[CIT0130] Lichti NI, Steele MA, Swihart RK 2017 Seed fate and decision-making processes in scatter-hoarding rodents. Biological Reviews of the Cambridge Philosophical Society 92:474–504. 26587693 10.1111/brv.12240

[CIT0131] Lichti NI, Steele MA, Zhang H, Swihart RK 2014 Mast species composition alters seed fate in North American rodent-dispersed hardwoods. Ecology 95:1746–1758. 25163109 10.1890/13-1657.1

[CIT0132] Liehrmann O, Jégoux F, Guilbert MA, Isselin-Nondedeu F, Saïd S, Locatelli Y, Baltzinger C 2018 Epizoochorous dispersal by ungulates depends on fur, grooming and social interactions. Ecology and Evolution 8:1582–1594. 29435234 10.1002/ece3.3768 PMC5792512

[CIT0133] Lisci M, Pacini E 1997 Fruit and seed structural characteristics and seed dispersal in *Mercurialis annua* L. (Euphorbiaceae). Acta Societatis Botanicorum Poloniae 66:379–386.

[CIT0134] Lotan A, Izhaki AL 2013 Could abiotic environment shape fleshy fruit traits? A field study of the desert shrub *Ochradenus baccatus*. Journal of Arid Environments 92:34–41.

[CIT0135] Lu JJ, Ma WB, Tan DY, Baskin JM, Baskin CC 2013 Effects of environmental stress and nutlet morph on proportion and within-flower number-combination of morphs produced by the fruit-dimorphic species *Lappula duplicicarpa* (Boraginaceae). Plant Ecology 214:351–362.

[CIT0136] Luna CA, Loayza AP, Squeo FA 2016 Fruit size determines the role of three scatter-hoarding rodents as dispersers or seed predators of a fleshy-fruited Atacama Desert shrub. PLoS One 11:e0166824. 27861550 10.1371/journal.pone.0166824 PMC5115819

[CIT0270] Manasse RS. 1990 Seed size and habitat effects on the water-dispersed perennial, Crinum erubescens (Amaryllidaceae). American Journal of Botany 77:1336–1341.

[CIT0137] Manasse RS, Howe HF 1983 Competition for dispersal agents among tropical trees: influences of neighbors. Oecologia 59:185–190. 28310232 10.1007/BF00378836

[CIT0138] Mandák B, Pyšek P 1999 Effects of plant density and nutrient levels on fruit polymorphism in *Atriplex sagittata*. Oecologia 119:63–72. 28308160 10.1007/s004420050761

[CIT0139] Manzaneda AJ, Rey PJ, Alcántara JM 2009 Conflicting selection on diaspore traits limits the evolutionary potential of seed dispersal by ants. Journal of Evolutionary Biology 22:1407–1417. 19460082 10.1111/j.1420-9101.2009.01752.x

[CIT0140] Marchetto KM, Shea K, Kelly D, Groenteman R, Sezen Z, Jongejans E 2014 Unrecognized impact of a biocontrol agent on the spread rate of an invasive thistle. Ecological Applications 24:1178–1187. 25154105 10.1890/13-1309.1

[CIT0141] Markl JS, Schleuning M, Forget PM, Jordano P, Lambert JE, Traveset A, Wright SJ, Böhning-Gaese K 2012 Meta-analysis of the effects of human disturbance on seed dispersal by animals. Conservation Biology 26:1072–1081. 22971077 10.1111/j.1523-1739.2012.01927.x

[CIT0142] Martínez I, García D, Obeso JR 2007 Allometric allocation in fruit and seed packaging conditions the conflict among selective pressures on seed size. Evolutionary Ecology 21:517–533.

[CIT0143] Martínez I, González-Taboada F 2009 Seed dispersal patterns in a temperate forest during a mast event: performance of alternative dispersal kernels. Oecologia 159:389–400. 19018573 10.1007/s00442-008-1218-4

[CIT0144] Martorell C, Martínez-López M 2014 Informed dispersal in plants: *Heterosperma pinnatum* (Asteraceae) adjusts its dispersal mode to escape from competition and water stress. Oikos 123:225–231.

[CIT0145] McArthur C, Orlando P, Banks PB, Brown JS 2012 The foraging tightrope between predation risk and plant toxins: a matter of concentration. Functional Ecology 26:74–83.

[CIT0146] McCabe JD, Olsen BJ 2015 Tradeoffs between predation risk and fruit resources shape habitat use of landbirds during autumn migration. The Auk 132:903–913.

[CIT0271] McKey D. 1975 The ecology of coevolved seed dispersal systems. In: Gilbert LE, Raven PH, eds. Coevolution of animals and plants. Austin: University of Texas Press, 159–191.

[CIT0147] Meyer SE, Carlson SL 2001 Achene mass variation in *Ericameria nauseosus* (Asteraceae) in relation to dispersal ability and seedling fitness. Functional Ecology 15:274–281.

[CIT0148] Michaels HJ, Benner B, Hartgerink AP, Lee TD, Rice S, Willson MF, Bertin RI 1988 Seed size variation: magnitude, distribution, and ecological correlates. Evolutionary Ecology 2:157–166.

[CIT0149] Monty A, Maebea L, Mahya G, Brown CS 2016 Diaspore heteromorphism in the invasive *Bromus tectorum* L. (Poaceae): sterile florets increase dispersal propensity and distance. Flora 224:7–13.

[CIT0150] Morales JM, Rivarola MD, Amico G, Carlo TA 2012 Neighborhood effects on seed dispersal by frugivores: testing theory with a mistletoe-marsupial system in Patagonia. Ecology 93:741–748. 22690625 10.1890/11-0935.1

[CIT0151] Moran EV, Clark JS 2012 Between-site differences in the scale of dispersal and gene flow in red oak. PLoS One 7:e36492. 22563504 10.1371/journal.pone.0036492 PMC3341347

[CIT0152] Morán-López T, Alonso CL, Díaz M 2015a Landscape effects on jay foraging behavior decrease acorn dispersal services in dehesas. Acta Oecologica 69:52–64.

[CIT0153] Morán-López T, Carlo TA, Amico G, Morales JM 2018a Diet complementation as a frequency-dependent mechanism conferring advantages to rare plants via dispersal. Functional Ecology 32:2310–2320.

[CIT0154] Morán‐López T, Fernández M, Alonso CL, Flores‐Rentería D, Valladares F, Díaz M 2015b Effects of forest fragmentation on the oak–rodent mutualism. Oikos 124:1482–1491.

[CIT0155] Morán-López T, Valladares F, Tiribelli F, Pérez-Sepúlveda JE, Traveset A, Díaz M 2018b Fragmentation modifies seed trait effects on scatter-hoarders’ foraging decisions. Plant Ecology 219:325–342.

[CIT0156] Moreira JI, Riba-Hernández P, Lobo JA 2017 Toucans (*Ramphastos ambiguus*) facilitate resilience against seed dispersal limitation to a large-seeded tree (*Virola surinamensis*) in a human-modified landscape. Biotropica 49:502–510.

[CIT0157] Morse DH, Schmitt J 1985 Propagule size, dispersal ability, and seedling performance in *Asclepias syriaca*. Oecologia 67:372–379. 28311571 10.1007/BF00384943

[CIT0158] Muhanguzi HDR, Ipulet P 2012 Fruiting phenology of fig trees in Kalinzu Forest, Uganda. African Journal of Ecology 50:90–101.

[CIT0159] Muñoz A, Bonal R 2007 Rodents change acorn dispersal behaviour in response to ungulate presence. Oikos 116:1631–1638.

[CIT0160] Muñoz A, Bonal R 2008a Are you strong enough to carry that seed? Seed size/body size ratios influence seed choices by rodents. Animal Behaviour 76:709–715.

[CIT0161] Muñoz A, Bonal R 2008b Seed choice by rodents: learning or inheritance? Behavioral Ecology and Sociobiology 62:913–922.

[CIT0162] Nathan R 2006 Long-distance dispersal of plants. Science 313:786–788. 16902126 10.1126/science.1124975

[CIT0163] Nathan R, Safriel UN, Noy-Meir I 2001 Field validation and sensitivity analysis of a mechanistic model for tree seed dispersal by wind. Ecology 82:374–388.

[CIT0164] Norghauer JM, Newbery DM 2015 Tree size and fecundity influence ballistic seed dispersal of two dominant mast-fruiting species in a tropical rain forest. Forest Ecology and Management 338:100–113.

[CIT0165] Norghauer JM, Nock CA, Grogan J 2011 The importance of tree size and fecundity for wind dispersal of big-leaf mahogany. PLoS One 6:e17488. 21408184 10.1371/journal.pone.0017488 PMC3049789

[CIT0166] Obeso JR, Martínez I, García D 2011 Seed size is heterogeneously distributed among destination habitats in animal dispersed plants. Basic and Applied Ecology 12:134–140.

[CIT0167] Ohsawa T, Tsuda Y, Saito Y, Sawada H, Lde Y 2007 Steep slopes promote downhill dispersal of *Quercus crispula* seeds and weaken the fine-scale genetic structure of seedling populations. Annals of Forest Science 64:405–412.

[CIT0168] Ortiz-Pulido R, Albores-Barajas YV, Díaz SA 2007 Fruit removal efficiency and success: influence of crop size in a neotropical treelet. Plant Ecology 189:147–154.

[CIT0169] Palacio FX, Lacoretz M, and Ordano M 2014 Bird-mediated selection on fruit display traits in *Celtis ehrenbergiana* (Cannabaceae). Evolutionary Ecology Research 16:51–62.

[CIT0170] Palacio FX, Ordano M 2018 The strength and drivers of bird-mediated selection on fruit crop size: a meta-analysis. Frontiers in Ecology and Evolution 6:18.

[CIT0171] Palacio FX, Valoy M, Bernacki F, Sánchez MS, Núñez-Montellano MG, Varela O, Ordano M 2017 Bird fruit consumption results from the interaction between fruit-handling behaviour and fruit crop size. Ethology Ecology & Evolution 29:24–37.

[CIT0172] Pan Y, Bai B, Xiong T, Shi P, Lu C 2016 Seed handling by primary frugivores differentially influence post-dispersal seed removal of Chinese yew by ground-dwelling animals. Integrative Zoology 11:191–198. 26846724 10.1111/1749-4877.12189

[CIT0173] Parciak W 2002 Environmental variation in seed number, size, and dispersal of a fleshy-fruited plant. Ecology 83:780–793.

[CIT0174] Paulsen TR, Colville L, Kranner I, Daws MI, Högstedt G, Vandvik V, Thompson K 2013 Physical dormancy in seeds: a game of hide and seek? The New Phytologist 198:496–503. 23421728 10.1111/nph.12191

[CIT0175] Paulsen TR, Högstedt G, Thompson K, Vandvik V, Eliassen S, Leishman M 2014 Conditions favouring hard seededness as a dispersal and predator escape strategy. The Journal of Ecology 102:1475–1484. 25558091 10.1111/1365-2745.12323 PMC4277852

[CIT0176] Pearse IS, Koenig WD, Kelly D 2016 Mechanisms of mast seeding: resources, weather, cues, and selection. The New Phytologist 212:546–562. 27477130 10.1111/nph.14114

[CIT0177] Perea R, López D, San Miguel A, Gil L 2012 Incorporating insect infestation into rodent seed dispersal: better if the larva is still inside. Oecologia 170:723–733. 22588632 10.1007/s00442-012-2350-8

[CIT0178] Pesendorfer MB, Koenig WD 2016 The effect of within-year variation in acorn crop size on seed harvesting by avian hoarders. Oecologia 181:97–106. 26809620 10.1007/s00442-016-3557-x

[CIT0179] Pesendorfer MB, Koenig WD 2017 Competing for seed dispersal: evidence for the role of avian seed hoarders in mediating apparent predation among oaks. Functional Ecology 31:622–631.

[CIT0180] Pesendorfer MB, Sillett TS, Morrison SA, Kamil AC 2016 Context-dependent seed dispersal by a scatter-hoarding corvid. The Journal of Animal Ecology 85:798–805. 26876417 10.1111/1365-2656.12501

[CIT0181] Pizo MA, Almeida-Neto M 2009 Determinants of fruit removal in *Geonoma pauciflora*, an understory palm of neotropical forests. Ecological Research 24:1179–1186.

[CIT0182] Poulsen JR, Clark CJ, Connor EF, Smith TB 2002 Differential resource use by primates and hornbills: implications for seed dispersal. Ecology 83:228–240.

[CIT0183] Reid S, Armesto JJ 2011 Avian gut-passage effects on seed germination of shrubland species in Mediterranean central Chile. Plant Ecology 212:1–10.

[CIT0184] Rey PJ, Alcántara JM 2014 Effects of habitat alteration on the effectiveness of plant-avian seed dispersal mutualisms: consequences for plant regeneration. Perspectives in Plant Ecology, Evolution and Systematics 16:21–31.

[CIT0185] Rezvani M, Cousens RD, Zaefarian F, Karimmojeni K, Robinson AP 2010 Shapes of ballistic seed dispersal distributions: a comparison of *Oxalis corniculata* with a theoretical model. Weed Research 50:631–637.

[CIT0186] Roberts D, Ciuti S, Barber QE, Willier C, Nielsen SE 2018 Accelerated seed dispersal along linear disturbances in the Canadian oil sands region. Scientific Reports 8:4828. 29555925 10.1038/s41598-018-22678-y PMC5859175

[CIT0187] Rodríguez-Pérez J, Riera N, Traveset A 2005 Effect of seed passage through birds and lizards on emergence rate of Mediterranean species: differences between natural and controlled conditions. Functional Ecology 19:699–706.

[CIT0188] Rumeu B, Álvarez-Villanueva M, Arroyo JM, González-Varo JP 2019 Interspecific competition for frugivores: population-level seed dispersal in contrasting fruiting communities. Oecologia 190:605–617. 31197480 10.1007/s00442-019-04434-9

[CIT0189] Russo SE 2003 Responses of dispersal agents to tree and fruit traits in *Virola calophylla* (Myristicaceae): implications for selection. Oecologia 136:80–87. 12684855 10.1007/s00442-003-1239-y

[CIT0190] Sæther BE, Engen S 2015 The concept of fitness in fluctuating environments. Trends in Ecology & Evolution 30:273–281. 25843273 10.1016/j.tree.2015.03.007

[CIT0191] Sallabanks R 1993 Hierarchichal mechanisms of fruit selection by an avian frugivore. Ecology 74:1326–1336.

[CIT0192] SanMartin-Gajardo I, Morellato LP 2003 Inter and intraspecific variation on reproductive phenology of the Brazilian Atlantic forest Rubiaceae: ecology and phylogenetic constraints. Revista de Biologia Tropical 51:691–698. 15162775

[CIT0193] Saracco JF, Collazo JA, Groom MJ, Carlo TA 2005 Crop size and fruit neighborhood effects on bird visitation to fruiting *Schefflera morototoni* trees in Puerto Rico. Biotropica 37:81–87.

[CIT0194] Schaefer HM, Valido A, Jordano P 2014 Birds see the true colours of fruits to live off the fat of the land. Proceedings of the Royal Society of London B. Biological Sciences 281:20132516. 10.1098/rspb.2013.2516 PMC3896014 24403330

[CIT0195] Schmidt KA, Ostfeld RS 2003 Songbird populations in fluctuating environments: predator responses to pulsed resources. Ecology 84:406–415.

[CIT0196] Schreiber S, Beckman N 2019 Individual variation in dispersal and fecundity increases rates of spatial spread. AoB Plants, forthcoming. 10.1093/aobpla/plaa001 PMC7273335 32528638

[CIT0197] Schubert SC, Pesendorfer MB, Koenig WD 2018 Context-dependent post-dispersal predation of acorns in a California oak community. Acta Oecologica 92:52–58.

[CIT0198] Schupp EW 1988 Seed and early seedling predation in the forest understory and in treefall gaps. Oikos 51:71–78.

[CIT0199] Schupp EW 1993 Quantity, quality and the effectiveness of seed dispersal by animals. Vegetatio 107/108:15–29.

[CIT0200] Schupp EW 2007 The suitability of a site for seed dispersal is context-dependent. In: Dennis A, Green R, Schupp EW, Westcott D, eds. Seed dispersal: theory and its application in a changing world. Wallingford, UK: CAB International, 445–462.

[CIT0201] Schupp EW, Fuentes M 1995 Spatial patterns of seed dispersal and the unification of plant population ecology. Écoscience 2:267–275.

[CIT0202] Schupp EW, Jordano P, Gómez JM 2010 Seed dispersal effectiveness revisited: a conceptual review. The New Phytologist 188:333–353. 20673283 10.1111/j.1469-8137.2010.03402.x

[CIT0203] Schupp EW, Jordano P, Gómez JM 2017 A general framework for effectiveness concepts in mutualisms. Ecology Letters 20:577–590. 28349589 10.1111/ele.12764

[CIT0204] Schupp EW, Milleron T, Russo SE 2002 Dissemination limitation and the origin and maintenance of species–rich tropical forests. In: Levey DJ, Silva WR, Galetti M, eds. Seed dispersal and frugivory: ecology, evolution and conservation. Wallingford, UK: CAB International Publishing, 19–33.

[CIT0205] Shanahan M, Compton SG 2001 Vertical stratification of figs and fig-eaters in a Bornean lowland rain forest: how is the canopy different? Plant Ecology 153:121–132.

[CIT0206] Shave ME, Shwiff SA, Elser JL, Lindell CA 2018 Falcons using orchard nest boxes reduce fruit‐eating bird abundances and provide economic benefits for a fruit‐growing region. Journal of Applied Ecology 55:2451–2460.

[CIT0207] Sheldon JC, Burrows FM 1973 The dispersal effectiveness of the achene-pappus units of selected Compositae in steady winds with convection. New Phytologist 72:665–675.

[CIT0208] Shimada T, Takahashi A, Shibata M, Yagihashi T 2015 Effects of within-plant variability in seed weight and tannin content on foraging behaviour of seed consumers. Functional Ecology 29:1513–1521.

[CIT0209] Siepielski AM, Benkman CW 2008 A seed predator drives the evolution of a seed dispersal mutualism. Proceedings of the Royal Society of London B. Biological Sciences 275:1917–1925. 10.1098/rspb.2008.0451 PMC2593931 18460433

[CIT0210] Siepielski AM, Benkman CW 2010 Conflicting selection from an antagonist and a mutualist enhances phenotypic variation in a plant. Evolution 64:1120–1128. 19817846 10.1111/j.1558-5646.2009.00867.x

[CIT0211] Sinha A, Davidar P 1992 Seed dispersal ecology of a wind dispersed rain forest tree in the Western Ghats, India. Biotropica 24:519–526.

[CIT0212] Skarpaas O, Silverman EJ, Jongejans E, Shea K 2011 Are the best dispersers the best colonizers? Seed mass, dispersal and establishment in *Carduus* thistles. Evolutionary Ecology 25:155–169.

[CIT0213] Smith AD, McWilliams SR 2014 Fruit removal rate depends on neighborhood fruit density, frugivore abundance, and spatial context. Oecologia 174:931–942. 24305861 10.1007/s00442-013-2834-1

[CIT0214] Snell RS, Beckman NG, Fricke E, Loiselle BA, Carvalho CS, Jones LR, Lichti NI, Lustenhouwer N, Schreiber S, Strickland C, Sullivan LL, Cavazos BR, Giladi I, Hastings A, Holbrook K, Jongejans E, Kogan O, Montaño-Centellas F, Rudolph J, Rogers HS, Zwolak R, Schupp EW 2019 Consequences of intraspecific variation in seed dispersal for plant demography, communities, evolution and global change. AoB Plants 11:plz016; doi:10.1093/aobpla/plz016. 31346404 PMC6644487

[CIT0215] Sobral M, Guitián J, Guitián P, Larrinaga AR 2013 Selective pressure along a latitudinal gradient affects subindividual variation in plants. PLoS One 8:e74356. 24069297 10.1371/journal.pone.0074356 PMC3778006

[CIT0216] Sobral M, Larrinaga AR, Guitián J 2010 Do seed-dispersing birds exert selection on optimal plant trait combinations? Correlated phenotypic selection on the fruit and seed size of hawthorn (*Crataegus monogyna*). Evolutionary Ecology 24:1277–1290.

[CIT0217] Soons MB, Heil GW, Nathan R, Katul GG 2004 Determinants of long-distance seed dispersal by wind in grasslands. Ecology 85:3056–3068.

[CIT0218] Steele MA, Hadj-Chikh LZ, Hazeltine J 1996 Caching and feeding decisions by *Sciurus carolinensis*: responses to weevil-infested acorns. Journal of Mammalogy 77:305–314.

[CIT0219] Sunyer P, Muñoz A, Bonal R, Espelta JM 2013 The ecology of seed dispersal by small rodents: a role for predator and conspecific scents. Functional Ecology 27:1313–1321.

[CIT0272] Tackenberg O, Römermann C, Thompson K, Poschlod P 2006 What does diaspore morphology tell us about external animal dispersal? Evidence from standardized experiments measuring seed retention on animal-coats. Basic and Applied Ecology 7:45–58.

[CIT0220] Takagi E, Iguchi K, Suzuki M, Togashi K 2012 A seed parasitoid wasp prevents berries from changing their colour, reducing their attractiveness to frugivorous birds. Ecological Entomology 37:99–107.

[CIT0221] Takahashi A, Shibata M, Shimada T 2011 Variation in seed production schedule among individual trees of a deciduous oak species *Quercus serrata*: its relation to seed characteristics. Plant Ecology 212:1527–1535.

[CIT0222] Tella JL, Lambertucci SA, Speziale KL, Hiraldo F 2016 Large-scale impacts of multiple co-occurring invaders on monkey puzzle forest regeneration, native seed predators and their ecological interactions. Global Ecology and Conservation 6:1–15.

[CIT0223] Teller BJ, Campbell C, Shea K 2014 Dispersal under duress: can stress enhance the performance of a passively dispersed species? Ecology 95:2694–2698.

[CIT0224] Teller BJ, Marden JH, Shea K 2015 Covariation in abscission force and terminal velocity of windborne sibling seeds alters long-distance dispersal projections. Methods in Ecology and Evolution 6:593–599.

[CIT0225] Tewksbury JJ, Levey DJ, Huizinga M, Haak DC, Traveset A 2008 Costs and benefits of capsaicin-mediated control of gut retention in dispersers of wild chilies. Ecology 89:107–117. 18376552 10.1890/07-0445.1

[CIT0226] Thiede DA, Augspurger CK 1996 Intraspecific variation in seed dispersion of *Lepidium campestre*. American Journal of Botany 83:856–866.

[CIT0227] Thomson FJ, Letten AD, Tamme R, Edwards W, Moles AT 2018 Can dispersal investment explain why tall plant species achieve longer dispersal distances than short plant species? The New Phytologist 217:407–415. 28833231 10.1111/nph.14735

[CIT0228] Thomson FJ, Moles AT, Auld TD, Kingsford RT 2011 Seed dispersal distance is more strongly correlated with plant height than with seed mass. Journal of Ecology 99:1299–1307.

[CIT0229] Trakhtenbrot A, Katul GG, Nathan R 2014 Mechanistic modeling of seed dispersal by wind over hilly terrain. Ecological Modelling 274:29–40.

[CIT0230] Traveset A, Riera N, Mas RE 2001 Ecology of fruit-colour polymorphism in *Myrtus communis* and differential effects of birds and mammals on seed germination and seedling growth. Journal of Ecology 89:749–760.

[CIT0273] Traveset A, Roberstson A, Rodríguez-Pérez J 2007 A review on the role of endozoochory on seed germination. In: Dennis AJ, Schupp EW, Green RJ, Westcott DA, eds. Seed dispersal. Theory and its application in a changing world. Wallingford: CAB International, 78–103.

[CIT0231] Traveset A, Willson MF 1998 Ecology of the fruit-colour polymorphism in *Rubus spectabilis*. Evolutionary Ecology 12:331–345.

[CIT0232] Tsahar E, Friedman J, Izhaki I 2002 Impact on fruit removal and seed predation of a secondary metabolite, emodin, in *Rhamnus alaternus* fruit pulp. Oikos 99:290–299.

[CIT0233] Tutin CEG, Fernandez M 1994 Comparison of food processing by sympatric apes in the Lope Reserve, Gabon. In: Thierry B, Anderson JR, Roeder JJ, Herrenschmidt N, eds. Current primatology. Vol. I: ecology and evolution. Strasbourg, France: Louis Pasteur University, 29–36.

[CIT0234] Uriarte M, Anciães M, da Silva MT, Rubim P, Johnson E, Bruna EM 2011 Disentangling the drivers of reduced long-distance seed dispersal by birds in an experimentally fragmented landscape. Ecology 92:924–937. 21661555 10.1890/10-0709.1

[CIT0235] Vander Wall SB 2010 How plants manipulate the scatter-hoarding behaviour of seed-dispersing animals. Philosophical Transactions of the Royal Society of London. Series B, Biological Sciences 365:989–997. 20156821 10.1098/rstb.2009.0205 PMC2830241

[CIT0236] Vander Wall SB, Longland WS 2004 Diplochory: are two seed dispersers better than one? Trends in Ecology & Evolution 19:155–161. 16701247 10.1016/j.tree.2003.12.004

[CIT0237] Vanthomme H, Belle B, Forget PM 2010 Bushmeat hunting alters recruitment of large-seeded plant species in Central Africa. Biotropica 42:672–679.

[CIT0238] Wang B, Corlett RT 2017 Scatter‐hoarding rodents select different caching habitats for seeds with different traits. Ecosphere 8:e01774

[CIT0239] Wang B, Ives AR 2017 Tree-to-tree variation in seed size and its consequences for seed dispersal versus predation by rodents. Oecologia 183:751–762. 28000021 10.1007/s00442-016-3793-0

[CIT0240] Wang B, Yang X 2014 Teasing apart the effects of seed size and energy content on rodent scatter-hoarding behavior. PLoS One 9:e111389. 25350369 10.1371/journal.pone.0111389 PMC4211888

[CIT0241] Wang B, Yang X 2015 Seed removal by scatter-hoarding rodents: the effects of tannin and nutrient concentration. Behavioural Processes 113:94–98. 25625425 10.1016/j.beproc.2015.01.012

[CIT0242] Wang B, Ye C-X. Cannon CH, Chen J 2013 Dissecting the decision making process of scatter-hoarding rodents. Oikos 122:1027–1034.

[CIT0243] Weiblen GD, Thomson JD 1995 Seed dispersal in *Erythronium grandiflorum* (Liliaceae). Oecologia 102:211–219. 28306876 10.1007/BF00333253

[CIT0244] Wender NJ, Polisetty CR, Donohue K 2005 Density-dependent processes influencing the evolutionary dynamics of dispersal: a functional analysis of seed dispersal in *Arabidopsis thaliana* (Brassicaceae). American Journal of Botany 92:960–971. 21652479 10.3732/ajb.92.6.960

[CIT0245] Wenk EH, Falster DS 2015 Quantifying and understanding reproductive allocation schedules in plants. Ecology and Evolution 5:5521–5538. 27069603 10.1002/ece3.1802 PMC4813122

[CIT0246] Wessels S, Eichberg E, Storm C, Schwabe A 2008 Do plant-community-based grazing regimes lead to epizoochorous dispersal of high proportions of target species? Flora 203:304–326.

[CIT0247] Westcott DA 1994 Leks of leks: a role for hotspots in lek evolution? Proceedings of the Royal Society of London B. Biological Sciences 258:281–286.

[CIT0248] Westcott DA 1997 Lek locations and patterns of female movement and distribution in a Neotropical frugivorous bird. Animal Behaviour 53:235–247.

[CIT0249] Wheelwright NT 1993 Fruit size in a tropical tree species: variation, preference by birds, and heritability. Vegetatio 107/108:163–174.

[CIT0250] Whelan CJ, Schmidt KA, Steele BB, Quinn WJ, Dilger S 1998 Are bird-consumed fruits complementary resources? Oikos 83:105–205.

[CIT0251] Whitehead SR, Poveda K 2011 Herbivore-induced changes in fruit–frugivore interactions. Journal of Ecology 99:964–969.

[CIT0252] Whitney KD 2005 Linking frugivores to the dynamics of a fruit color polymorphism. American Journal of Botany 92:859–867. 21652467 10.3732/ajb.92.5.859

[CIT0274] Will SR, Maussner S, Tackenberg O 2007 Experimental studies of diaspore attachment to animal coat: predicting epizoochorous dispersal potential. Oecologia 153:331–339. 17516091 10.1007/s00442-007-0731-1

[CIT0253] Will H, Tackenberg O 2008 A mechanistic simulation model of seed dispersal by animals. Journal of Ecology 96:1011–1022.

[CIT0254] Willson MF, O'Dowd DJ 1989 Fruit color polymorphism in a bird-dispersed shrub (*Rhagodia parabolica*) in Australia. Evolutionary Ecology 3:40–50.

[CIT0255] Wróbel A, Zwolak R 2017 Deciphering the effects of disperser assemblages and seed mass on patterns of seed dispersal in a rodent community. Integrative Zoology 12:457–467. 28488781 10.1111/1749-4877.12265

[CIT0256] Wyse SV, Hulme PE, Holland EP 2019 Partitioning intraspecific variation in seed dispersal potential using a low‐cost method for rapid estimation of samara terminal velocity. Methods in Ecology and Evolution 10:1298–1307.

[CIT0257] Xiao Z, Zhang Z, Wang Y 2004 Dispersal and germination of big and small nuts of *Quercus serrata* in a subtropical broad-leaved evergreen forest. Forest Ecology and Management 195:141–150.

[CIT0258] Yadok BG, Gerhard D, Forget P-M, Chapman H 2018 Size doesn't matter: larger *Carapa* seeds are not dispersed farther by African rodent community. African Journal of Ecology 56:1028–1033.

[CIT0259] Yoshikawa T, Isagi Y, Kikuzawa K 2009 Relationships between bird-dispersed plants and avian fruit consumers with different feeding strategies in Japan. Ecological Research 24:1301–1311.

[CIT0260] Young LM, Kelly D 2018 Effects of seed dispersal and microsite features on seedling establishment in New Zealand fleshy-fruited perennial mountain plants. Austral Ecology 43:775–785.

[CIT0261] Zhang R, Gallagher RS, Shea K 2011 Maternal warming affects early life stages of an invasive thistle. Plant Biology 14:783–788. 10.1111/j.1438-8677.2011.00561.x 22404764

[CIT0262] Zhang H, Luo Y, Steele MA, Yang Z, Wang Y, Zhang Z 2013 Rodent-favored cache sites do not favor seedling establishment of shade-intolerant wild apricot (*Prunus armeniaca* Linn.) in northern China. Plant Ecology 214:531–543.

[CIT0263] Zhu J, Liu M, Xin Z, Zhao Y, Liu Z 2016 Which factors have stronger explanatory power for primary wind dispersal distance of winged diaspores: the case of *Zygophyllum xanthoxylon* (Zygophyllaceae)? Journal of Plant Ecology 9:346–356.

[CIT0264] Zungu MM, Downs CT 2015 Effects of tannins on fruit selection in three southern African frugivorous birds. Behavioural Processes 111:84–89. 25522953 10.1016/j.beproc.2014.12.003

[CIT0265] Zwolak R 2018 How intraspecific variation in seed-dispersing animals matters for plants. Biological Reviews of the Cambridge Philosophical Society 93:897–913. 29024277 10.1111/brv.12377

[CIT0266] Zwolak R, Bogdziewicz M, Wróbel A, Crone EE 2016 Advantages of masting in European beech: timing of granivore satiation and benefits of seed caching support the predator dispersal hypothesis. Oecologia 180:749–758. 26612728 10.1007/s00442-015-3511-3

